# Differential Gene Expression Patterns of EBV Infected EBNA-3A Positive and Negative Human B Lymphocytes

**DOI:** 10.1371/journal.ppat.1000506

**Published:** 2009-07-03

**Authors:** Marie L. Hertle, Claudia Popp, Sabine Petermann, Sabine Maier, Elisabeth Kremmer, Roland Lang, Jörg Mages, Bettina Kempkes

**Affiliations:** 1 Department of Gene Vectors, Helmholtz Center Munich, German Research Center for Environmental Health, Munich, Germany; 2 Institute of Molecular Immunology, Helmholtz Center Munich, German Research Center for Environmental Health, Munich, Germany; 3 Institute of Clinical Microbiology, Immunology and Hygiene, Friedrich-Alexander-University Erlangen-Nürnberg, Erlangen, Germany; 4 Institute of Medical Microbiology, Immunology and Hygiene, Technical University Munich, Munich, Germany; 5 Biotools B&M Labs, S.A., Madrid, Spain; Emory University, United States of America

## Abstract

The genome of Epstein-Barr virus (EBV) encodes 86 proteins, but only a limited set is expressed in EBV–growth transformed B cells, termed lymphoblastoid cell lines (LCLs). These cells proliferate via the concerted action of EBV nuclear antigens (EBNAs) and latent membrane proteins (LMPs), some of which are rate limiting to establish a stable homeostasis of growth promoting and anti-apoptotic activities. We show here that EBV mutants, which lack the EBNA-3A gene, are impaired but can still initiate cell cycle entry and proliferation of primary human B cells in contrast to an EBNA-2 deficient mutant virus. Surprisingly, and in contrast to previous reports, these viral mutants are attenuated in growth transformation assays but give rise to permanently growing EBNA-3A negative B cell lines which exhibit reduced proliferation rates and elevated levels of apoptosis. Expression profiles of EBNA-3A deficient LCLs are characterized by 129 down-regulated and 167 up-regulated genes, which are significantly enriched for genes involved in apoptotic processes or cell cycle progression like the tumor suppressor gene p16/INK4A, or might contribute to essential steps of the viral life cycle in the infected host. In addition, EBNA-3A cellular target genes remarkably overlap with previously identified targets of EBNA-2. This study comprises the first genome wide expression profiles of EBNA-3A target genes generated within the complex network of viral proteins of the growth transformed B cell and permits a more detailed understanding of EBNA-3A's function and contribution to viral pathogenesis.

## Author Summary

Epstein-Barr virus (EBV) infects primary human B cells and establishes a latent infection, which leads to permanently growing B cell cultures. These growth transformed B cells express a well defined set of latent viral genes, which are also expressed in post-transplant lymphomas of immunosuppressed patients. In a concerted action these latent viral proteins drive cellular proliferation and prevent apoptosis. For this study, recombinant Epstein-Barr virus mutants that lack the gene for the Epstein-Barr virus nuclear antigen-3A (EBNA-3A) were generated. EBNA-3A is a transcriptional modulator of gene expression. We show here that EBNA-3A deficient growth transformed B cells can be established *in vitro*. Our results suggest that EBNA-3A supports viability but is not absolutely essential for proliferation of the infected B cell. By virtue of the established EBNA-3A deficient cell lines, we could for the first time identify a broad array of cellular target genes controlled by EBNA-3A in EBV infected B cells. These EBNA-3A target genes will permit a more detailed understanding of EBNA-3A's function and contribution to viral pathogenesis.

## Introduction

Epstein-Barr virus (EBV) infection of resting primary B cells *in vitro* causes cell cycle entry of the infected cells, which convert into permanently proliferating lymphoblastoid cell lines (LCLs) by establishing a latent viral infection. Growth transformation of primary human B cells by EBV requires the concerted action of Epstein-Barr virus nuclear antigens (EBNAs) and latent membrane proteins (LMPs). The genes encoding the EBNA-3A, -3B and -3C proteins are tandemly arranged in the viral genome and share some regions of colinear homology. EBNA-3A belongs to the subgroup of latent viral proteins, which have been reported to be absolutely essential for the initial steps in the process of growth transformation and its maintenance in latently infected cells [1]. LCLs, which express a conditional EBNA-3A mutant, cease proliferation in the absence of functional EBNA-3A [2]. While these results firmly established that EBNA-3A significantly contributes to the maintenance of proliferation of LCLs, EBNA-3A negative LCLs have been described occasionally challenging the notion that viable EBNA-3A negative LCLs can be established [3],[4].

The EBNA-3A and -3C full length proteins score as transcriptional repressors in heterologous GAL4 dependent reporter gene assays. Protein fragments of both viral proteins exhibit repressive as well as activating functions [5],[6],[7]. Repression by EBNA-3A is dependent on the interaction with the co-repressor C-terminal-binding protein (CtBP), which can recruit HDAC activities and human Polycomb group proteins [8].

Importantly, EBNA-3A might be a competitive antagonist of the viral transactivator Epstein-Barr virus nuclear antigen-2 (EBNA-2), which is invariably co-expressed with EBNA-3A in LCLs. All EBNA-3 proteins bind to the cellular DNA-binding factor CBF1. CBF1 (C-promoter binding factor 1) is also known as RBP-Jκ or RBPJ and is a member of the CSL group of orthologues comprised of CBF1, Su(H) and Lag-1. CBF1 is a sequence specific DNA-binding protein, which recruits co-repressor complexes to regulatory elements of promoters. EBNA-2 can bind to CBF1, displace the co-repressor complex and activate transcription. EBNA-3 proteins can interfere with CBF1 dependent activation of the viral C- and LMP2A promoters by EBNA-2 in transient reporter gene assays [7],[9],[10],[11],[12],[13]. Since all EBNA transcripts can be driven by the viral C-promoter, the EBNA-3 proteins could be a component of an auto-regulatory feedback loop controlling their own expression. Importantly, EBNA-3A mutants deficient for binding to CBF1 or repression of C-promoter activation in reporter gene studies could not rescue the proliferation of LCLs lacking functional EBNA-3A [14].

Transcriptional activation of EBNA-2 cellular target genes including *c-myc*, CD21 and CD23 can also be affected by EBNA-3A, if EBNA-3A is strongly expressed in LCLs. This finding indicated that EBNA-2 and EBNA-3A might be antagonists competing for access to cellular targets via CBF1 [15]. However, conditional inactivation of EBNA-3A in LCLs does not affect *c-myc* or CD23 expression indicating that expression levels are critical for EBNA-3A functions [2].

Anti-apoptotic functions have been attributed to the expression of EBNA-3 proteins in Burkitt's lymphoma cell lines and most recently it has been shown that EBNA-3A and -3C cooperate to repress the expression of the pro-apoptotic tumor suppressor Bim [16],[17]. EBNA-3A can also induce the chaperones Hsp40 and Hsp70 and co-chaperon Bag3 by a CtBP independent mechanism [18]. By directly interacting with the aryl-hydrocarbon receptor (AHR) EBNA-3A enhances the activity of this transcription factor [19]. However, cellular target genes which are modulated by this mechanism have not been described. In summary, several molecular strategies might be employed by EBNA-3A to modulate gene expression and cell survival but information on *bona fide* target genes is rare, since most targets have been defined by reporter gene assays or ectopic overexpression of EBNA-3A.

In order to re-assess the contribution of EBNA-3A to the growth transformation process, we generated EBNA-3A negative recombinant virus and infected resting human B cells *in vitro*. Surprisingly, EBNA-3A deficient virus infected human primary B cells entered the cell cycle. Compared to EBNA-3A positive control cultures, they expand more slowly and exhibit elevated steady state levels of apoptosis. Permanently growing EBNA-3A negative LCLs could be established and were used to identify the contribution of EBNA-3A to the transcription program of the EBV infected B cell.

## Results

### EBNA-3A negative viral mutants have impaired growth transformation properties compared to wild-type controls but give rise to permanently growing B cell cultures

In order to re-asses the contribution of EBNA-3A to the B cell growth transformation process we generated EBNA-3A deficient recombinant B95.8 mutants, termed EBV-E3AmtA and EBV-E3AmtB, based on the 2089 homologous recombination system in *E.coli*, also called Maxi-EBV [20]. Recombinant B95.8 (2089) will be designated EBVwt in the following. Previously published EBNA-3A deficient viral mutants carried truncations of the open reading frame at aminoacid position 302 or 304, respectively [1],[4]. EBV-E3AmtA was an insertion mutant disrupting the open reading frame of EBNA-3A at aminoacid position 126 of the primary sequence. In addition, a second mutant, EBV-E3AmtB, was generated in which the entire open reading frame of EBNA-3A was deleted.

**Figure 1:**
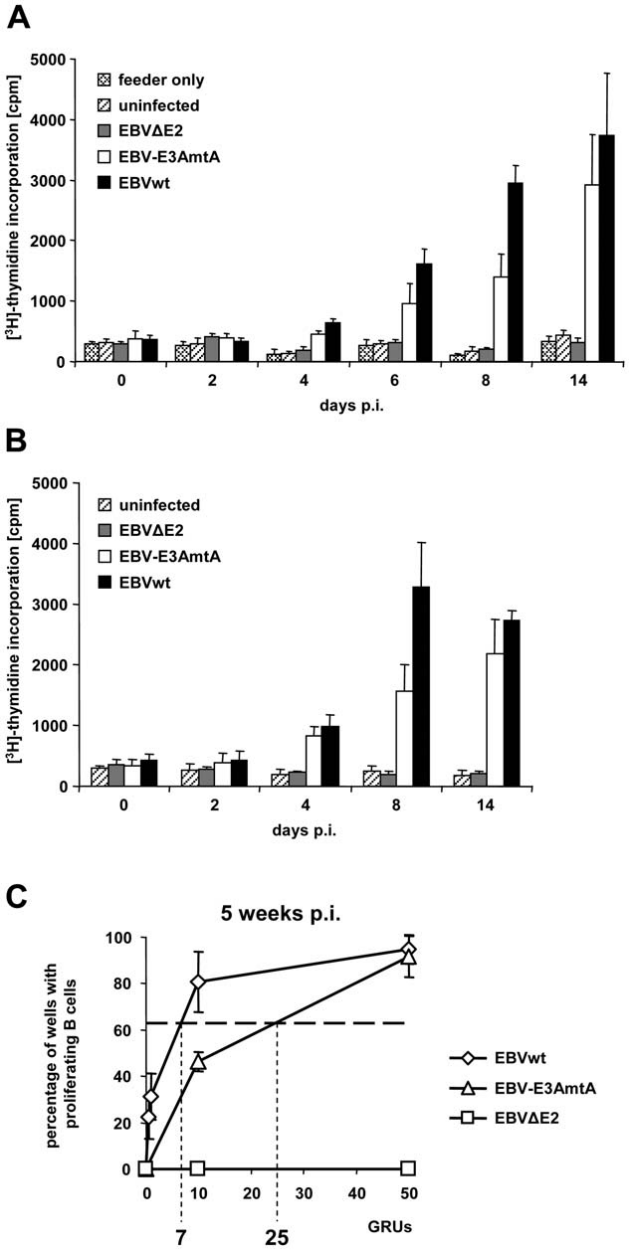
EBNA-3A negative viral mutants drive cell cycle entry of primary human B cells but show reduced long-term growth transformation capacity compared to EBVwt. (A) Cell cycle entry of primary human B cells after infection with EBVwt and EBV-E3AmtA was analyzed by thymidine incorporation assays. 2×10^5^ B cells were plated on lethally irradiated MRC5 feeder layer and infected with 3000 GRUs of EBVwt, EBV-E3AmtA or EBVΔE2 or left uninfected. At day 0, 2, 4, 6, 8 and 14 p.i. cells were pulsed with [^3^H]-thymidine and analyzed for thymidine incorporation. Cultures with feeder only were set up in parallel and show the background levels of [^3^H]-thymidine measurements. Results are given as means from 6 single values and represent one of three independent experiments. (B) 2×10^5^ B cells derived from the same donor as analyzed in (A) were plated without feeder cells and infected with 3000 GRUs of EBVwt, EBV-E3AmtA or EBVΔE2 or left uninfected. At day 0, 2, 4, 8 and 14 p.i. cells were pulsed with [^3^H]-thymidine and analyzed for thymidine incorporation. Results are given as means from 6 single values. (C) The growth transformation efficiency of EBV-E3AmtA is weakly impaired but not abolished. To assess the growth transformation capacity of EBV-E3AmtA, the number of GRUs required to sustain B cell proliferation in a single well of a 96-well cluster plate was determined for EBVwt and EBV-E3AmtA in limiting dilution assays. Briefly, primary B cells derived from 4 individual donors were infected with serial dilutions of normalized viral supernatants and plated on lethally irradiated MRC5 feeder layer in groups of 48 cultures per degree of dilution. The percentage of wells per group with proliferating cells was determined 5 weeks p.i. with EBVwt and EBV-E3AmtA. The results are given as the mean percentage of proliferating cultures per group and the standard deviations are shown as error bars. The horizontal line at 63% (30 out of 48 wells plated) indicates the zero term of the Poisson equitation and identifies the average number of GRUs necessary to establish one proliferating B cell culture. Control infections with EBVΔE2 were set up in parallel for each donor but never gave rise to proliferating cultures.

In order to analyze whether EBNA-3A negative viruses can promote the activation and S-phase entry of human primary B cells, CD19 positive B cells were purified from adenoids. These B cells were infected with viral supernatants of EBV-E3AmtA and plated on lethally irradiated human primary fibroblasts as feeder layers. For comparison, EBVwt and non-transforming EBV mutants lacking the EBNA-2 ORF (EBVΔE2) were analyzed in parallel (Figure 1A). In order to measure the earliest time point at which the cells entered S-phase, 2×10^5^ CD19 positive B cells were infected with 3000 Green Raji Units (GRUs) of viral supernatants and [^3^H]-thymidine incorporation was measured immediately after virus infection on day 0 and on day 2, 4, 6, 8, and 14 post infection (p.i.). To define the background levels of the biological system, thymidine measurements of irradiated feeder layers and of uninfected B cells plated on feeder layers were also tested in parallel cultures. Thymidine incorporation of cultures infected with both EBVwt and EBV-E3AmtA initiated on day 4 and was readily seen on day 6 p.i. and the following days of culture. EBV-E3AmtA was less efficient than EBVwt infection to drive proliferation during a two weeks time period. Infection with EBVΔE2 never gave rise to thymidine incorporating cultures. Similar results were obtained in parallel studies, using the same donor material but performed in the absence of fibroblast feeder layers (Figure 1B). Since cell cultures set up at low cell densities might be more feeder dependent, we performed thymidine incorporation experiments using 1×10^5^ or 0.5×10^5^ cells per microculture (Figure S1). Cell cycle entry of EBVwt infected B cells was unaffected by fibroblast support. However, co-cultivated fibroblasts strongly promoted cell cycle entry of EBV-E3AmtA infected B cells in low density cell cultures which still scored below wt levels (Figure S1). Hence, B cells were co-cultivated with irradiated fibroblast feeder layers for the initial 28–35 days p.i. with EBVwt or mutant viruses in all subsequent experiments since we wanted to exclude all potentially limiting factors caused by unfavorable conditions during prolonged cell culture.

Limiting dilutions of viral supernatants were used to measure the relative efficiency of EBV-E3AmtA compared to EBVwt to give rise to permanently growing cultures *in vitro* (Figure 1C). 1×10^5^ B cells isolated from adenoids of 4 donors were plated in groups of 48 microcultures on lethally irradiated fibroblast feeder layers. The microcultures were infected with serial dilutions of normalized viral supernatants (2000, 1000, 200, 100, 50, 10, 1, 0.5 GRUs per well). 5 weeks p.i. proliferating cultures were counted. On average, 7 EBVwt GRUs were required to establish one proliferating B cell culture. The growth transformation efficiency of EBV-E3AmtA was about 3.6 fold reduced and required 25 GRUs to initiate and maintain a proliferating culture for 5 weeks (Figure 1C). Noteworthy, proliferation of EBNA-3A negative cultures was not only restricted to this limited time period, since long term B cell lines negative for EBNA-3A could be established from these cultures for all 4 donors (designated as donor 4, 5, 6 and 7 in the following).

In order to generate further long term B cell cultures infected with EBNA-3A negative virus, CD19 positive B cells were isolated from 3 additional donors and infected with EBVwt, EBV-E3AmtA and EBV-E3AmtB. From each donor infected with EBNA-3A negative viruses permanently growing B cell cultures could be established, which proved to be of B cell origin by FACS analysis for CD19 expression (Figure S2). In contrast, EBVΔE2 infected B cell cultures never gave rise to transiently or permanently growing B cell cultures.

**Figure 2:**
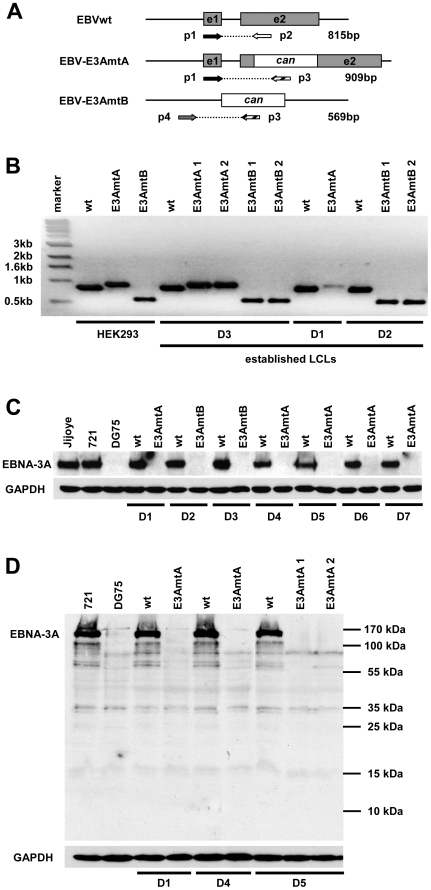
EBNA-3A negative viral mutants give rise to permanently growing lymphoblastoid B cell lines. (A) Diagram of the EBNA-3A gene locus in the EBVwt genome and the two distinct recombinant viral mutants. In order to generate EBV-E3AmtA a canamycin resistance gene (can) cassette was inserted into the second exon of EBNA-3A, while the entire coding sequence of EBNA-3A was replaced by a canamycin cassette in order to generate EBV-E3AmtB. The position of primers (p) used for analysis of the EBNA-3A locus in wt or modified EBV genomes and the expected product sizes are depicted. (B) LCLs established from 3 individual donors (D1–D3) by infection of B cells with EBVwt, EBV-E3AmtA or EBV-E3AmtB virus stocks and the corresponding HEK293 virus producing helper cell lines were tested for the correct state of the modified EBNA-3A gene locus by PCR. (C) EBNA-3A negative LCLs are not co-infected with EBV type II. Wt and EBNA-3A negative LCLs established from 7 individual donors (D1–D7) were analyzed for EBNA-3A expression by immunoblotting using a polyclonal α-EBNA-3A antibody detecting both, EBV type I and II encoded EBNA-3A. The EBV type II infected cell line Jijoye, the EBV type I infected cell line 721, and the EBV-negative cell line DG75 were included as controls. (D) The disruption of the EBNA-3A ORF by the canamycin cassette does not lead to expression of a truncated EBNA-3A protein. LCLs established by infection with EBV-E3AmtA were inspected for expression of a truncated EBNA-3A protein by immunoblotting using a monoclonal α-EBNA-3A antibody detecting an epitope within the first 50 amino acids of EBNA-3A. The respective wt LCLs, the EBV-positive cell line 721 and the EBV-negative cell line DG75 were included as controls. GAPDH immunodetection was used to control for equal loading of the lanes.

By PCR analysis the genotype of established B cell lines from donor 1, 2 and 3 infected with EBVwt, EBV-E3AmtA and EBV-E3AmtB was verified (Figure 2A and B). The PCR products generated from genomic DNA of HEK293 producer cell lines were identical in size to those generated from B cell lines infected with the corresponding viruses. The genotype of the infected B cell lines was further confirmed by genomic southern blots (Figure S3).

In order to exclude that proliferation of EBNA-3A negative cultures is driven by co-infection with EBV type II, EBNA-3A protein expression was analyzed in western blots using an EBNA-3A specific polyclonal sheep serum, which recognizes EBNA-3A of the EBV strain type I, B95.8, as well as the EBNA-3A protein of the EBV strain type II, Jijoye. Neither type I nor II derived EBNA-3A protein was detected in B cell cultures infected with EBNA-3A negative viruses (Figure 2C). For all 7 donors co-infection with EBV type II was further excluded by PCR analysis using primers specific for EBV type I and II genomic sequences, respectively (Figure S4).

Since the EBV-E3AmtA virus carries a disrupted EBNA-3A gene, which might encode for a truncated protein of 126 aminoacids, western blot experiments were carried out in order to visualize this potential EBNA-3A fragment (Figure 2D). A rat monoclonal antibody, which recognizes an epitope within the first 50 aminoacids of EBNA-3A (E3AN4A5), was used for immunostaining in western blots. This antibody readily detected full length EBNA-3A in EBVwt infected B cells but no truncated EBNA-3A fragment was detected in EBV-E3AmtA infected cultures. If a potential EBNA-3A fragment is made in EBV-E3AmtA infected cells it is expressed below detection levels.

### EBNA-3A negative LCLs proliferate at reduced rates, show elevated levels of apoptotic death rates but exhibit viral gene expression patterns similar to EBVwt infected cells

**Figure 3:**
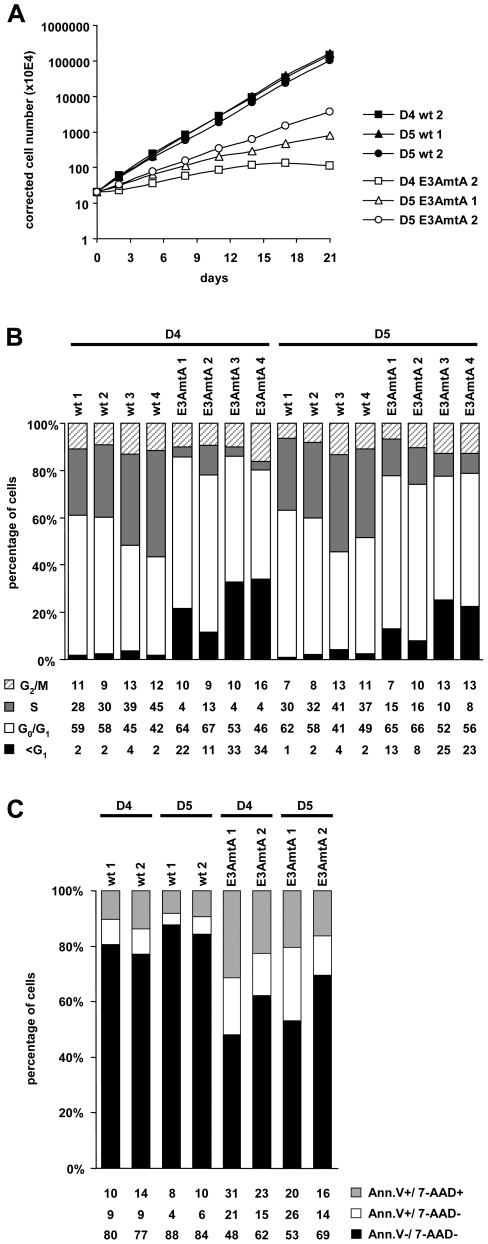
EBNA-3A negative LCLs proliferate at reduced rates and exhibit higher levels of apoptotic cells. (A) EBNA-3A negative LCLs proliferate at reduced rates. Three independent wt and EBNA-3A negative LCLs derived from two individual donors were seeded at an initial density of 2×10^5^ cells per ml and viable cell counts were determined over a period of three weeks. Results are given as total numbers of viable cells corrected for the expansion of the cultures over time. The data are shown as mean values of triplicates. (B) EBNA-3A negative LCLs show reduced S-phase entry compared to wt LCLs. The cell cycle status of wt and EBNA-3A negative LCLs derived from two different donors was determined with the thymidine analogue BrdU, which was added to the respective cultures for 2 hrs prior to FACS analysis. The incorporated BrdU was stained with an APC-coupled anti-BrdU antibody and total DNA was counterstained with 7-AAD. Cells were inspected for G_0_/G_1_, S and G_2_/M phases of the cell cycle and for sub G1 DNA content by FACS analysis. (C) EBNA-3A negative LCLs exhibit higher levels of apoptotic cells compared to wt LCLs. The fraction of apoptotic cells in cultures of wt and EBNA-3A negative LCLs was determined by FACS analysis after staining of cells with Cy5-coupled Annexin V and 7-AAD. Cells that stain positive for Annexin V-Cy5 but negative for 7-AAD are in early apoptosis, while cells that stain positive for both are either in the end stage of apoptosis or dead.

EBNA-3A negative LCLs could be established from all donors tested so far and were expanded in the absence of feeder layers 4–5 weeks post infection. However, EBNA-3A negative cell lines were difficult to expand and viability was impaired compared to wt LCLs. In order to characterize their phenotype in more detail, established EBNA-3A negative cultures, which had been cultivated for at least 2.5 months but not longer than 4 months p.i. were tested for their proliferation rates. Compared to wt control lines isolated from identical donors EBV-E3AmtA infected cells proliferated at reduced rates (Figure 3A). The relative distribution of cells in the different phases of the cell cycle was analyzed after BrdU pulse labeling for two hours and 7-AAD counter staining followed by FACS analysis (Figure 3B). In EBV-E3AmtA infected B cell lines the relative proportion of S-phase cells was decreased, the G1 population was slightly increased, while the apoptotic sub G1 population was strongly increased. In order to further quantify the dead and apoptotic cell fractions, cells were stained with Annexin V and 7-AAD (Figure 3C). Both, early apoptotic (Annexin V +/7-AAD −) as well as late stage apoptotic and dead (Annexin V +/7-AAD +) cells were enriched in EBV-E3AmtA infected cell cultures, indicating that the impaired proliferation rates of the cultures are caused by a steady loss of cells by apoptosis.

Since the EBNA-3A deficient phenotype might reflect a greater dependence on autocrine/paracrine factors for growth and survival, proliferation rates of EBNA-3A negative cultures were further tested in the presence of cell culture supernatants derived from wt LCLs or fibroblast feeder layers. However, no improvement in proliferation rates could be detected within a time period of 15 days (Figure S5A and B). In addition, EBNA-3A negative LCLs were co-cultivated on fibroblast feeder layers for 3 days, but again the impaired growth of the cells could not be restored (Figure S5C). Hence, fibroblast feeder cells might support the growth of EBNA-3A negative cultures in the initial phase of growth transformation, but cannot revert the EBNA-3A deficient growth phenotype of established LCLs. If EBNA-3A negative LCLs were cultivated for periods extending 4 months p.i., the poorer growth and survival phenotype was gradually lost and cultures adopted growth characteristics similar to wt LCLs.

**Figure 4:**
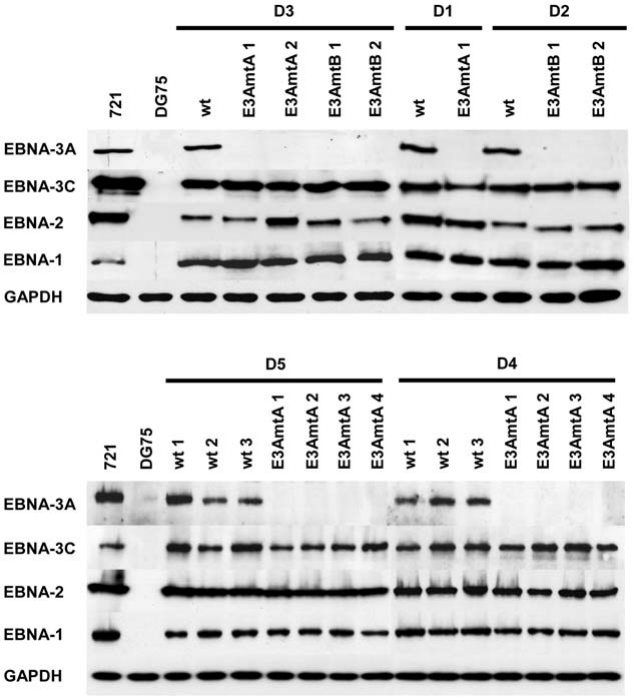
EBNA-3A negative LCLs exhibit protein expression levels of EBV nuclear antigens similar to wt LCLs. Wt and EBNA-3A negative LCLs derived from 5 individual donors were analyzed for expression levels of EBNA-3A, -3C, -2 and -1 by immunoblotting. The EBV-positive cell line 721 and the EBV-negative cell line DG75 were used as positive and negative control, respectively. GAPDH immunodetection was used to control for equal loading of the lanes.

**Figure 5:**
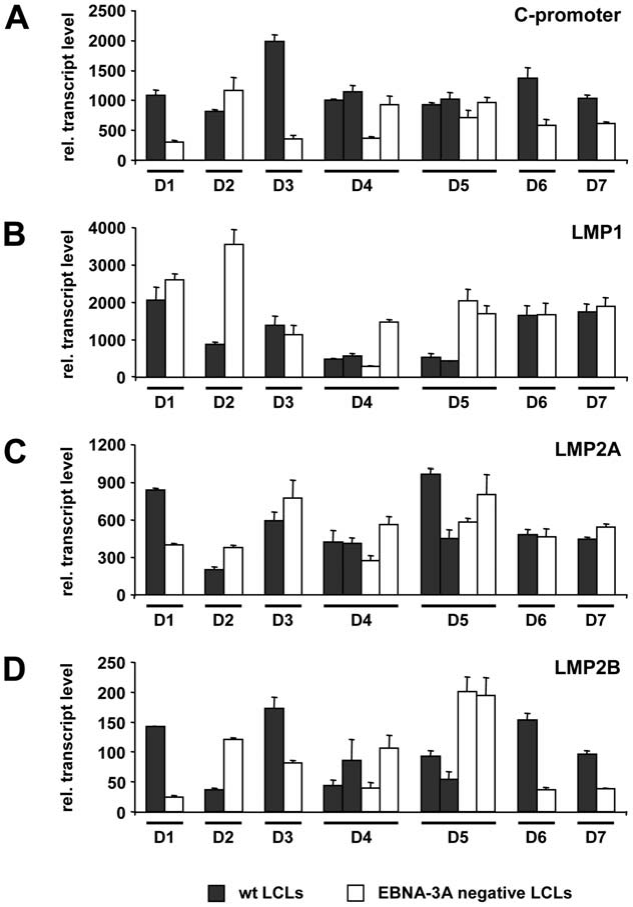
EBV infected B cells show no EBNA-3A dependent changes in C-promoter usage and expression patterns of latent membrane proteins. LCLs established from 7 different donors by infection of B cells with EBVwt or either EBV-E3AmtB (D2, D3) or EBV-E3AmtA (D1, D4–D7) were analyzed for (A) C-promoter usage and transcript levels of (B) LMP1, (C) LMP2A and (D) LMP2B by real-time RT-PCR. Mean values of triplicates are expressed as relative units after internal normalization for 18S rRNA levels.

To further characterize EBNA-3A negative LCLs, the expression of latent viral genes typically expressed in EBV growth transformed B cells was investigated (Figure 4 and 5). All EBNAs, which have been shown to be critical for the growth transformation process, EBNA-1, -2, and -3C, were consistently expressed at wild-type levels (Figure 4).

The viral C-promoter (Cp) is considered to be a central promoter element of all EBNAs in the latency III transcription program. Real-time RT-PCR for C-promoter usage was performed for EBNA-3A negative LCLs of all 7 donors aiming at identifying potential changes in Cp usage compared to EBVwt infected cells (Figure 5A). However, no consistent EBNA-3A dependent alterations in Cp usage could be found. Similarly, no consistent change of LMP1, LMP2A or LMP2B transcript abundance could be seen in EBNA-3A negative LCLs (Figure 5B, C, D).

### Genome wide analysis of cellular genes differentially expressed by EBNA-3A positive and negative LCLs reveals a remarkable overlap of EBNA-3A and EBNA-2 target genes

To identify potential differential cellular gene expression patterns of EBNA-3A negative LCLs, total cellular RNA was harvested and processed for hybridization to HG-U133A 2.0 Affymetrix arrays. This array carries 22,277 independent probe sets, which represent 18,400 transcripts and related variants, including 14,500 well characterized human genes. The samples were prepared from 5 EBVwt, 5 EBV-E3AmtA and 4 EBV-E3AmtB infected LCLs derived from 3 individual donors, which had been cultivated for 3 (D3), 4 (D2) and 6 (D1) months post infection. At the time the RNA was harvested these LCLs had been routinely expanded in the absence of feeder cells for at least 2 and up to 5 months but still exhibited the characteristics of wt and EBNA-3A deficient growth phenotypes.

**Figure 6:**
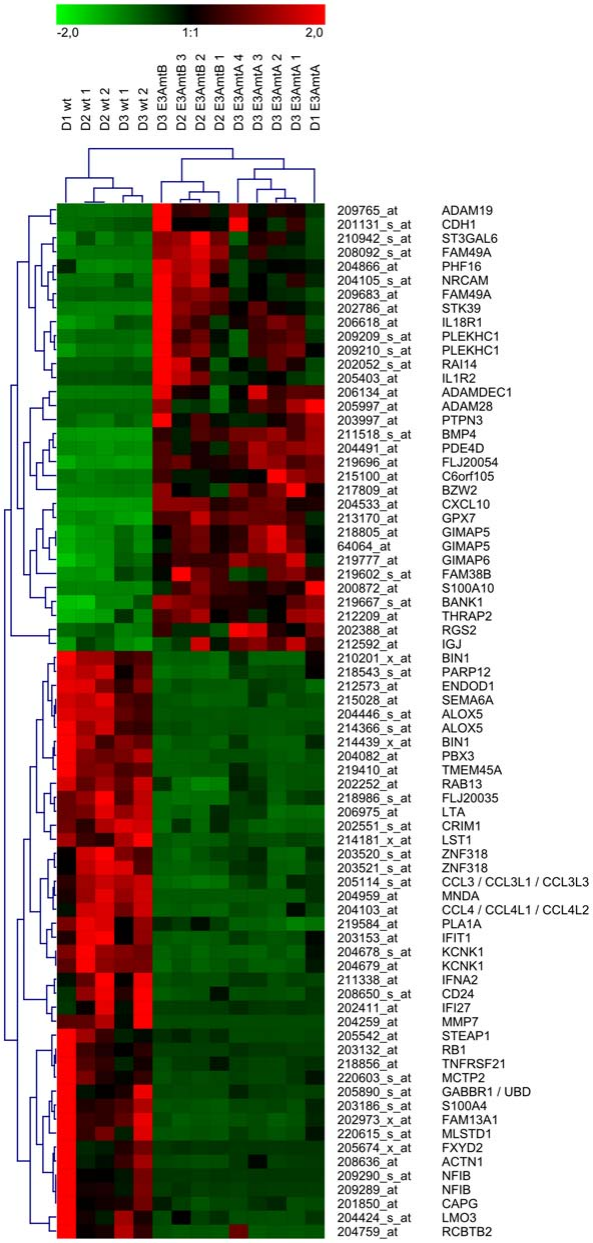
Expression profiles of wt and EBNA-3A negative LCLs. Shown are 74 probe sets displaying at least 4-fold changes in expression levels with significance p≤0.01 in EBNA-3A negative LCLs compared to wt LCLs. Vertical columns represent data obtained for each individual cell line by hybridization to a single microarray, while horizontal rows represent data obtained for a particular probe set across all cell lines. After normalization of expression values on a scale ranging from −2.0 to 2.0 for each probe set, an unsupervised hierarchical clustering analysis was performed, using Pearson correlation as a measure for similarity between genes and complete linkage as a clustering allocation algorithm. High expression values are represented by red, low expression values by green and medium values by black.

43.5% (+/−1.8%) of the probe sets scored present in EBVwt, EBV-E3AmtA and EBV-E3AmtB infected cells indicating that no massive over all change in transcription levels had occurred. Probe sets for CD antigens typically expressed on LCLs (CD19, CD20, CD21, CD23, CD40, CD86) scored present in both, EBNA-3A positive and negative LCLs, but were not differentially expressed (data not shown). Probe sets representing CD antigens expressed on T cells like CD3 or on specific B cell subsets like CD5 scored absent in all cell lines assayed. 380 probe sets, corresponding to 296 genes and 3 not yet annotated loci were differentially expressed at least 2-fold (p≤0.05) comparing wt and EBNA-3A negative LCLs. Of these 296 genes, 129 genes were found to be down-regulated in wt LCLs, while 167 genes were found to be up-regulated (see Table S1). In order to visualize differential gene expression patterns characteristic for EBNA-3A positive and negative cells, unsupervised hierarchical clustering of expression values was performed for 74 probe sets which exhibited ≥4-fold differential expression (p≤0.01) between the two groups (Figure 6). The result grouped samples according to the presence (lane 1–5) or absence of EBNA-3A (lane 6–14) expression and identified two main clusters of 29 EBNA-3A repressed and 37 EBNA-3A induced genes. EBV-E3AmtA and EBV-E3AmtB infected cells separate into two distinct but closely related clusters. The unsupervised hierarchical clustering for genes differentially expressed at least 2-fold (p≤0.05) is shown in Figure S6.

Based on the group of genes regulated by EBNA-3A at least 4-fold (p≤0.01), a subset of genes was selected for validation. Transcript levels of the selected target genes were analyzed by real-time RT-PCR in donor matched paired RNA samples of EBNA-3A positive and EBNA-3A negative LCLs derived from 3 individual donors (Table 1 and 2). For these experiments novel RNA preparations were used. LCLs from one donor (D3) were identical to those, which had been examined by microarray hybridization (designated as D3 wt 1 and D3 E3AmtB in Figure 6) and represent long-term established cell lines. In contrast, LCLs from donors 6 and 7 (D6, D7) were harvested 6 weeks p.i. with EBVwt or EBV-E3AmtA and thus represent recently established LCLs. All 12 genes, which had been identified as EBNA-3A repressed genes according to the Affymetrix analysis, could be confirmed in all three RNA pairs. Numerically, x-fold repression rates as measured by real-time RT-PCR in almost all cases exceeded the values obtained by the Affymetrix technology.

**Table 1:** Confirmation of Affymetrix data by real-time RT-PCR for cellular target genes repressed by EBNA-3A.

Gene symbol/aliases (Entrez Gene ID^a^)	Gene name	Molecular or biological function^b^	Affymetrix^c^	RT-PCR D3^d^	RT-PCR D6^e^	RT-PCR D7^e^
ADAMDEC1 (27299)	ADAM-like protein decysin 1	disintegrin metalloproteinase; cell adhesion	44.8	>135^f^	>55^f^	>80^f^
CXCL10 (3627)	Chemokine (C-X-C motif) ligand 10	CXCR3 ligand, chemotattractant for monocyctes, NK- and T-cells; inflammatory response	30.2	42.0	834.3	208.4
BMP4 (652)	Bone morphogenetic protein 4	growth factor; differentiation, osteogenesis	17.5	155.9	>200^f^	153.3
BZW2 (28969)	Basic leucine zipper and W2 domain-containing protein 2	cell differentiation	13.4	11.7	29.0	27.1
STK39 (27347)	Serine threonine kinase 39 (STE20/SPS1 homolg, yeast)	response to hypotonic stress	13.0	>240^f^	>110^f^	>80^f^
S100A10 (6281)	S100 calcium binding protein A10	annexin II ligand; signal transduction, membrane trafficking	12.2	66.0	36.3	41.5
FAM49A (81553)	Family with sequence similarity 49, member A	unknown	12.1	42.0	>20^f^	>15^f^
FLJ20054/DENND1B (163486)	hypothetical protein FLJ20054/DENN/MADD domain containing 1B	unknown	12.0	14.2	7.7	4.3
CDH1 (999)	Cadherin 1, type 1, E-cadherin (epithelial)	calcium dependent cell-cell adhesion glycoprotein	7.8	35.6	40.4	16.5
PDE4D (5144)	Phosphodiesterase 4D, cAMP-specific	regulation of intracellular cAMP level	6.6	10.7	6.2	7.4
PLEKHC1/FERMT2 (10979)	Pleckstrin homology domain-containing family C member 1 fermitin family homolog 2	actin cytoskeleton organization, cell adhesion	6.5	14.2	5.7	5.3
GIMAP5 (55340)	GTPase, IMAP family member 5	mitochondrial integrity	5.8	2.0	16.0	6.7

a Unique gene identification number (ID) according to the National Center for Biotechnology Information (NCBI).

b As defined by GO-term and/or Refseq (NCBI).

c x-fold repression was calculated from mean expression values of wt and EBNA-3A negative LCLs originating from the Affymetrix gene array hybridization.

d x-fold repression according to real-time RT-PCR data was calculated from mean expression values of 2 independent RNA samples derived from a wt and an EBNA-3A negative LCL, respectively, which were established from donor 3. Note that LCLs from donor 3 were already examined by the Affymetrix screen and represent long-term established B cell lines.

e x-fold repression according to real-time RT-PCR data was calculated from mean expression values of 2 independent RNA samples derived from a wt and an EBNA-3A negative LCL, respectively, which were established from donors 6 and 7. Note that LCLs from donors 6 and 7 represent freshly established LCLs and were analyzed 6 weeks p.i..

f minimum x-fold repression was estimated for genes that were down-regulated to levels below detection limits by setting the value to a minimal transcript level detected for the gene.

**Table 2:** Confirmation of Affymetrix data by real-time RT-PCR for cellular target genes induced by EBNA-3A.

Gene symbol/aliases (Entrez Gene ID^a^)	Gene name	Molecular or biological function^b^	Affymetrix^c^	RT-PCR D3^d^	RT-PCR D6^e^	RT-PCR D7^e^
MMP7 (4316)	Matrix metalloproteinase 7 (matrilysin)	breakdown of extracellular matrix	49.4	>160^f^	>30^f^	>70^f^
ALOX5 (240)	Arachidonate 5-lipoxygenase	leukotriene biosynthesis, inflammatory response	36.7	>30^f^	3.6	5.1
RB1 (5925)	Retinoblastoma-associated protein	tumor suppressor gene; cell cycle regulation, heterochromatin formation	8.9	10.0	4.8	5.9
S100A4/MTS1 (6275)	S100 calcium binding protein A4/metastasin	cell motility, invasion, tubulin polymerization	8.7	12.3	1.6	2.5
TMEM45A (55076)	Transmembrane protein 45a	unknown	7.8	7.7	1.4	2.8
PBX3 (5090)	Pre-B cell leukemia homeobox 3	transcription factor	7.5	7.9	5.1	6.1
LTA (4049)	Lymphotoxin alpha (TNF superfamily, member 1)	cytokine; signal transduction, inflammatory response	7.4	33.5	1.4	1.4
CCL3 (6348)	Chemokine (C-C motif) ligand 3	lymphocyte trafficking	5.9	6.6	2.1	2.2
BIN1 (274)	Myc box-dependent-interacting protein 1 (bridging integrator 1)	cell proliferation	5.6	19.3	3.1	5.5
LST1 (7940)	Leukocyte-specific transcript 1 protein	immune response	5.4	7.7	1.6	2.1
FAM13A1 (10144)	Family with sequence similarity 13, member A1	unknown	5.2	6.4	1.8	1.9
MLSTD1/FAR2 (55711)	Male sterility domain-containing protein 1/Fatty acyl-CoA reductase 2	oxidoreductase, lipid metabolism	5.1	>30^f^	2.7	3.0

a Unique gene identification number (ID) according to the National Center for Biotechnology Information (NCBI).

b As defined by GO-term and/or Refseq (NCBI).

c x-fold induction was calculated from mean expression values of wt and EBNA-3A negative LCLs originating from the Affymetrix gene array hybridization.

d x-fold induction according to real-time RT-PCR data was calculated from mean expression values of 2 independent RNA samples derived from a wt and an EBNA-3A negative LCL, respectively, which were established from donor 3. Note that LCLs from donor 3 were already examined by the Affymetrix screen and represent long-term established B cell lines.

e x-fold induction according to real-time RT-PCR data was calculated from mean expression values of 2 independent RNA samples derived from a wt and an EBNA-3A negative LCL, respectively, which were established from donors 6 and 7. Note that LCLs from donors 6 and 7 represent freshly established LCLs and were analyzed 6 weeks p.i..

f minimum x-fold induction was estimated for genes that were up-regulated from levels below detection limits by setting the value to a minimal transcript level detected for the gene.

12 genes, which had scored as at least 4-fold induced genes according to the array analysis, could all be confirmed for the donor 3 derived RNA samples of the long-term established LCLs. However, with the exception of MMP7, induction rates were significantly lower but still detectable, when recently established LCLs were analyzed. Some of the EBNA-3A activated genes are differentially expressed in long term lines but only marginally modulated in lines 6 weeks p.i. (TMEM45A, LTA, LST1, FAM13A1). Most likely, for these genes the differential expression is triggered by indirect mechanisms including the selective pressure imposed by the lack of EBNA-3A in the cell culture. In order to test if expression profiles of EBNA-3A deficient LCLs were robust, the transcript levels of all 24 genes were re-assessed in a cell line, which had been continuously propagated for more than 2 years and now grew with wild-type characteristics. Of all genes tested, the expression levels of Cadherin 1 (CDH1) only had dropped to levels measured in the corresponding EBNA-3A proficient cell line (data not shown). Further experiments will be required to clarify whether the loss of CDH1 expression *in vitro* has provided a growth advantage to the cells. Interestingly, 5 (CXCL10, FAM49A, CDH1, ALOX5, CCL3) of the selected 24 EBNA-3A targets have already been shown to be regulated by EBNA-2 before. Since EBNA-3A and EBNA-2 have been suggested to balance CBF1 signaling we analyzed whether further previously identified EBNA-2 target genes were represented in the set of the 296 EBNA-3A regulated genes (see Table S1). EBNA-2 targets were selected from a set of 505 genes, which were regulated at least 2-fold (p≤0.05) by EBNA-2 in EBV negative B cell lines and had been identified by Affymetrix gene array analysis previously [21]. In addition, published EBNA-2 targets from different sources [22],[23] were included in the analysis. Apparently, counter- and co-regulated genes were identified. On the one hand, 26 of 129 EBNA-3A repressed genes were induced by EBNA-2 and 22 of 167 EBNA-3A induced genes were repressed by EBNA-2. Hence 48 of the 296 EBNA-3A targets (16.2%) are counter-regulated by EBNA-2. On the other hand, 9 of 129 EBNA-3A repressed genes were also repressed by EBNA-2, while 18 of 167 induced target genes were also induced by EBNA-2. Thus, 27 out of 296 EBNA-3A targets (9.1%) are co-regulated by EBNA-2. Taken together, it appears that EBNA-3A and EBNA-2 indeed regulate an overlapping set of target genes, while the frequency of counter-regulated genes appears to be slightly elevated compared to co-regulated genes. In summary, a total of 25.3% of EBNA-3A target genes are also affected by EBNA-2 strongly suggesting that both viral proteins are functionally linked impinging on similar regulatory elements.

### EBNA-3A cellular target genes are enriched for genes contributing to cell survival

Given the unaltered viral gene expression patterns in EBNA-3A negative LCLs, we next asked if changes in cellular target gene expression might reflect functions of EBNA-3A, which explain the impaired viability of the EBNA-3A negative LCLs. A gene ontology (GO) analysis was performed with the set of genes with at least 2-fold (p≤0.05) expression changes comparing wt and EBNA-3A negative LCLs.

The online tool *DAVID* (**D**atabase for **A**nnotation, **V**isualization and **I**ntegrated **D**iscovery) was used to map EBNA-3A target genes to GO-terms in the “Biological Process” category and to calculate the significance for enrichment of specific GO-terms within this gene list with respect to the total number of genes assayed and annotated. Statistical measures for specific enrichment were assigned by means of an EASE score, a modified Fisher Exact p-value. Genes involved in an “immune system process” obtained the most significant scores (Table 3). However, this finding might be considered as less meaningful, since transcriptional profiling might simply reflect the B cell origin of the LCLs. Not surprisingly, genes annotated to immune system processes were also significantly enriched among EBNA-2 targets identified in the B cell lines BJAB and BL41 in a previous Affymetrix gene array analysis [21]. Noteworthy, genes annotated to be involved in “apoptosis” were strongly over-represented among EBNA-3A targets (35 members; EASE = 7.4E-5). Enrichment of the term “apoptosis” was absent among randomly selected groups of 380 reference probe sets as well as among EBNA-2 targets identified in BJAB cells conditional for EBNA-2 [21]. The group of EBNA-3A regulated genes annotated to “apoptosis” contained both anti-apoptotic genes as well as pro-apoptotic genes. For instance, wt LCLs exhibit higher expression levels of NOL3 (synonym ARC, apoptosis repressor with CARD domain; 2.0-fold induced, p<0.01) and BIRC3 (synonym cIAP2, inhibitor of apoptosis protein 2 homologue C; 2.1-fold induced, p<0.01) compared to EBNA-3A negative LCLs. Both proteins were repeatedly shown to inhibit apoptosis [24],[25, and references therein]. Concomitantly, expression of the pro-apoptotic genes PERP (TP53 apoptosis effector; 5.7-fold repressed, p = 0.02) and STK17B (synonym DRAK2, DAP kinase-related apoptosis-inducing protein kinase 2; 3.1-fold repressed, p = 0.02) [26],[27] appears to be repressed by EBNA-3A in wt LCLs. Significant enrichment was also found for genes annotated to be involved in the “regulation of cell cycle” (24 members; EASE = 1.3E-3). Prominent members within this group are e.g. cyclin D2 (2.0-fold induced, p = 0.02), which is up-regulated in wt LCLs, and the CDKN2A locus (2.7-fold repressed, p<0.01), which appears to be down-regulated in wt compared to EBNA-3A negative LCLs (for details see Table S1). The CDKN2A locus encodes the two tumor suppressor proteins p14/ARF and p16/INK4A. According to the Affymetrix data, further cyclin-dependent kinase inhibitors were not differentially expressed (p18, p21, and p57) or not expressed (p15 and p19). Transcript levels of CDKN1B, encoding p27, were significantly (p = 0.03) but only 1.5-fold reduced in wt compared to EBNA-3A negative LCLs (data not shown).

**Table 3:** GO-term enrichment analysis of cellular genes displaying at least 2-fold changes in expression levels between wt and EBNA-3A negative LCLs (p≤0.05).

GO-term^a^	Count^b^	EASE score^c^
immune system process	54	9.8E-13
response to virus	12	8.0E-6
chemotaxis	14	1.6E-5
apoptosis	35	7.4E-5
cell differentiation	61	9.8E-5
regulation of cell cycle	24	1.3E-3
signal transduction	87	1.6E-3
cell proliferation	29	1.0E-2

a GO-term in the “Biological Process” category. In order to highlight major biological themes and to reduce redundancy, similar annotation terms (e.g. “apoptosis” and “programmed cell death”) were grouped together and representative terms were manually selected. Note that a given gene can be annotated to multiple terms, since the gene ontology is structured as a directed acyclic graph.

b number of EBNA-3A target genes annotated to the term.

c statistical measure for enrichment of a given GO-term among differentially regulated genes with respect to the total number of genes assayed and annotated to the term. Functional groups enriched within EBNA-3A target genes were considered as meaningful in case of EASE-scores≤0.01.

**Figure 7:**
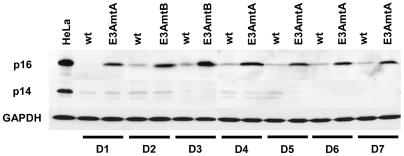
EBNA-3A negative LCLs exhibit higher expression levels of p16. Total cellular protein extracts of EBNA-3A positive and negative LCLs derived from 7 donors were analyzed for p14 and p16 protein expression by western blot analysis. HeLa cell extracts served as positive control for both proteins. GAPDH immunodetection was used to control for equal loading of the lanes.

Differential expression of p16 was already demonstrated for LCLs with a conditional EBNA-3C allele [28]. Since the probe sets for the CDKN2A locus cannot distinguish between the transcripts encoding for p14 and p16, wt and EBNA-3A negative LCLs from all seven donors were investigated for p14 and p16 expression by western blot analysis (Figure 7). Indeed, as described for EBNA-3C depleted LCLs, EBNA-3A negative LCLs exhibit higher levels of p16 expression. Expression of p14 was near or below detection levels. Hence, prevention of p16 expression in wt LCLs seems to be a common feature of both viral proteins, EBNA-3A as well as EBNA-3C.

## Discussion

### EBNA-3A deficient LCLs can be established with moderate efficiencies but are less viable than EBNA-3A proficient LCLs

Since the growth transformation process is based on the concerted action of viral proteins, the rate-limiting and distinct contribution of each viral factor can only be fully recognized in the cellular background of EBV infected B cells. For this study, recombinant EBV mutants deficient for EBNA-3A expression were generated and shown to be able to promote proliferation and viability.

Comparative limiting dilution analysis using mutant and wild-type virus revealed that the growth transformation efficiency of EBNA-3A deficient EBV was reduced 3 to 4-fold but permanently growing B cell cultures could be expanded from every donor sample that was infected.

Since we could establish long-term proliferating LCLs deficient for EBNA-3A from seven unrelated donors, we can exclude that genetic donor-specific features permitted the proliferation of the cells in our experiments. Importantly, we excluded the contribution of a potentially co-infecting endogenous donor derived virus of either type I or II providing EBNA-3A functions *in trans* by western blot and PCR analysis. These EBNA-3A deficient proliferating cultures are prototypic CD19 positive B cells, which can be propagated indefinitely *in vitro* and exhibit all features, which characterize an LCL. In addition, their gene expression profiles do not reveal any specific features, which would characterize them as specific B cell subsets.

Our findings appear to contradict previous studies, which concluded that EBNA-3A is essential for B cell growth transformation [1],[2],[4],[14]. An inherent feature of two earlier experimental approaches was the fact, that recombinant virus was produced with low efficiencies in the presence of excess EBNA-3A proficient helper virus [1],[4]. Infection with EBNA-3A deficient virus never generated proliferating cultures, which were initially EBNA-3A negative. For both previous studies either B cells isolated from peripheral blood or cord blood lymphocytes were infected. In the present study, we used purified B cell preparations from adenoids, which might represent a B cell population that is pre-activated and thus might growth transform more readily. Also, in the study performed by Tomkinson and colleagues, infected B cells were not co-cultivated with irradiated feeder layers as it has been done in this study. Although we can show, that EBNA-3A deficient established LCLs can be cultivated in the absence of feeders our data strongly suggest that fibroblast feeder layers stabilize the culture under suboptimal conditions and thus supported the growth transformation process. Thus, the optimized culture conditions as well as the B cell source might have well attributed to our recent findings. In addition, Tomkinson and colleagues reported that the inactivation of the EBNA-3A gene by insertional mutagenesis at aminoacid position 302 could have resulted in a dominant negative mutant since wild-type virus transformation, if performed in the presence of the co-infecting EBNA-3A mutant, was drastically reduced [1]. Thus, expression of a potentially toxic EBNA-3A fragment could have prevented the outgrowth of EBNA-3A deficient LCLs. Our previous studies, which worked on EBNA-3A mutants potentially encoding 304 aminoacids of EBNA-3A, revealed that LCLs, which were initially co-infected with the EBNA-3A proficient P3HR1 plus the EBNA-3A mutant could lose the P3HR1 genome over prolonged cell culture time, indicating that EBNA-3A is not required for maintenance of proliferation but a contribution to initiation could not be excluded [4]. Remarkably, the group of Robertson reported that they isolated a spontaneously growing LCL, which carried an EBV genome with a 16 kb deletion encompassing the EBNA-3A open reading frame and was thus negative for EBNA-3A. Since the deletion also included genes critical for the lytic life cycle, it most likely occurred post infection [3]. The fact that we reproducibly can establish EBNA-3A deficient LCLs is thus completely novel. Since for this study EBNA-3A was interrupted at aminoacid position 126 and a corresponding EBNA-3A fragment could not be detected in western blots, we suggest that our knock-down strategy could be one explanation for the apparently contradicting results to earlier experimental approaches. Notably, primary B cells could also be growth transformed using EBV-E3AmtB, which lacks the entire open reading frame of EBNA-3A. Since EBV-E3AmtB virus production was consistently lower than EBV-E3AmtA virus production, all quantitative experiments were performed using EBV-E3AmtA. On average, 25 “Green Raji Units” of EBV-E3AmtA virus stocks were sufficient to initiate a proliferating LCL culture. We suspect that the synchronous outgrowth of multiple clones within a single microculture well favors the survival of EBNA-3A negative LCLs by mutual cross feeding culture conditions. In summary, technical improvements during the last years, in particular the option that biological active titres of the viral supernatants can be quantified and normalized, might explain why previous attempts to raise these cultures were unsuccessful.

A recent study using conditional EBNA-3A mutant cell lines, which require functional EBNA-3A for proliferation, convincingly showed that EBNA-3A is necessary for growth maintenance in a conditional cellular system but contradicts the main result of our study [2]. We suggest the two distinct experimental approaches most likely account for this discrepancy.

Viral gene expression patterns were not altered in EBNA-3A deficient LCLs. We cannot exclude that a selective pressure caused by the EBNA-3A deficiency in our system forced the expression patterns of some viral factors. However, the published conditional system elegantly avoids this problem and confirms our results. Hence, viral gene expression levels are unlikely to account for the impaired viability of EBNA-3A deficient LCLs.

Even if EBNA-3A deficient LCLs can be propagated *in vitro*, their impaired viability most likely prevents the expansion of EBNA-3A deficient cells *in vivo*. Several convincing lines of evidence point towards an essential contribution of EBNA-3A to viral pathogenesis in the infected host *in vivo*. Infectious EBNA-3A negative clinical virus isolates have never been reported and so far only a single spontaneous EBNA-3A negative LCL was described [3]. In addition, under selective pressure imposed by adoptive immunotherapy targeting EBNA-3A, EBNA-3A loss of function mutants as described for EBNA-3B have never been observed [29].

### Gene expression profiles of EBNA-3A proficient and deficient LCLs

The gene expression profiles described in this study are based on the analysis of 5 EBNA-3A proficient LCLs compared to 9 EBNA-3A deficient LCLs. The RNA samples were isolated from three unrelated donors and data were later confirmed by further experiments using material from 2 additional unrelated donors. Since EBNA-3A is a transcriptional repressor in GAL4 based reporter gene assays, our target screen aimed primarily at identifying cellular genes specifically repressed by EBNA-3A in LCLs. However, according to the unsupervised clustering of the array results, EBNA-3A proficient and deficient LCLs express significantly distinct transcript profiles of 129 down-regulated but also of 167 up-regulated genes (≥2-fold, p≤0.05). By real-time RT-PCR we confirmed 24 either up- or down-regulated transcripts using RNAs from long term or recently established cell lines. Confirmation of the EBNA-3A repressed genes in general reflected the amplitude of change seen in the array data for long as well as for short term established lines. EBNA-3A induced genes could be confirmed in long term established cell lines, but induction was less pronounced in recently established cell lines. Thus, secondary effects of prolonged cell culture periods might indirectly influence the expression of these genes.

HSPA6/7, BAG3, HSPA2, CDCP1, HSPA1A/1B/1L, HSPCA, SPHK1, DNAJA1, ITPR3, and CCL2 had been previously described to be regulated in response to adenovirus EBNA-3A in fibroblasts [18]. In EBNA-3A deficient LCLs we could not confirm regulation of these genes. Most likely, the experimental set up accounts for the observed discrepancies. In addition, the pro-apoptotic gene BCL2L11 (Bim) has been described to be repressed by EBNA-3A and EBNA-3C expression in the context of EBV negative Burkitt's lymphoma cell lines [17]. In LCLs BCL2L11 expression was low and no significant modulation was detected.

Recently, 3 EBNA-3B repressed (CXCR4, ENTH, TTF2), 3 EBNA-3C repressed (JAG1, NCALD, FLNA) and 2 EBNA-3C activated (ITGA4 and TCL1A) target genes were described. These studies were based on LCLs which either lacked EBNA-3B or lacked EBNA-3B combined with low level EBNA-3C expression [30]. We found 3 of the genes repressed either by EBNA-3B or -3C also significantly repressed by EBNA-3A (NCALD, CXCR4, and ENTH). None of the activated genes was also modulated by EBNA-3A. In addition, suppression of p16/INK4A expression in the presence of functional EBNA-3C has recently been shown in an EBNA-3C conditional system [28] and was now also shown for EBNA-3A in our study. Thus, EBNA-3A and EBNA-3C indeed might share a set of target genes for which each EBNA-3 protein is rate limiting since loss of one EBNA-3 protein relieves repression. Hence, non-redundant functions can be executed by individual EBNA-3 proteins even in the context of single target genes. In addition to these targets, specific target genes exist, which are controlled by distinct EBNA-3 proteins.

Since EBNA-2 and all EBNA-3 proteins are co-expressed in LCLs and interact with CBF1, the EBNA-3 proteins might not only directly repress target gene expression but could also indirectly affect EBNA-2 functions. Several scenarios have been proposed. Since it is well established that EBNA-3 proteins interfere with CBF1 dependent EBNA-2 activation in reporter gene assays, the EBNA-3 proteins could directly compete for binding of EBNA-2 to CBF1 complexed with DNA [31]. Alternatively the EBNA-3 proteins could prevent CBF1/DNA complex formation and serve as buffers to bind CBF1 in solution and render it biologically unavailable for EBNA-2 [7],[32]. Intriguingly, the viral LMP1 promoter is co-activated by EBNA-3C and EBNA-2, and both proteins have been shown to bind to the endogenous LMP1 promoter indicating that on the promoter level EBNA-2 and -3C binding is not mutually exclusive in specific constellations [33],[34],[35]. However, the cooperation of EBNA-3C and EBNA-2 is PU.1 dependent and not executed by EBNA-3A [36]. While the LMP1 promoter is a valuable model system to study EBNA-3C/EBNA-2 interactions, no such model system is available for EBNA-3A. Since no endogenous viral latent gene was modulated by EBNA-3A, an important aspect of our study was to compare the set of EBNA-3A targets to previously identified EBNA-2 target genes in order to identify potential co- or counter-regulated cellular genes. Like the study published by Maruo and colleagues, our data neither confirmed antagonistic activities of EBNA-2 and EBNA-3A for the viral LMP2A and C-promoter nor for the *c-myc*, CD21 and CD23 gene reported previously [2],[9],[11],[15]. However, our analysis showed that a total of 16.2% of the EBNA-3A targets were counter-regulated by EBNA-2, while 9.1% were co-regulated genes. For instance, the cyclin-dependent kinase 5, regulatory subunit 1 (CDK5R1) and the chemokine ligand 3 (CCL3, MIP-1α) are activated by EBNA-2 via a CBF1 dependent mechanism [21] but are either down- (CDK5R1) or up-regulated (CCL3) by EBNA-3A. At this point of our study, we are confident that we have identified *bona fide* EBNA-3A regulated genes in the presence of the co-expressed EBNA-2 protein. Future studies will need to distinguish primary and secondary targets. We hope that our cellular system will facilitate the understanding of the molecular mechanism used by EBNA-2 and -3A to co-activate or counter-regulate cellular target genes by delineating cis-responsive regulatory elements of model target genes.

### Functions of EBNA-3A target genes

According to the GO term analysis, the biological functions of genes which are differentially expressed in EBNA-3A proficient versus deficient cells cover a broad spectrum ranging from immune system processes, chemotaxis, and apoptosis to cell cycle. It is to be expected that these EBNA-3A functions control the viability of the LCLs *in vitro* but also contribute to the viral life cycle and pathogenesis in the infected host.

The tumor suppressor gene p16/INK4A is a cell cycle inhibitor and senescence marker gene. A striking similarity of EBNA-3A and EBNA-3C negative LCLs is the expression of elevated p16/INK4A transcript and protein levels in both systems [28]. The 2-fold decrease of cyclin D2 in parallel with elevated p16/INK4A levels in EBNA-3A deficient LCLs are likely to impair the cell cycle progression. It would be interesting to know if the cell division cycle of a single cell deficient for EBNA-3A is prolonged. We are convinced that the constitutively elevated levels of apoptosis will necessarily cause a slower growth of the respective cultures but consider it most likely, that the modulation of cell cycle relevant genes imposes an additional constrain to the proliferating EBNA-3A deficient cell cultures. We thus do not anticipate that a single EBNA-3A target gene or a single class of genes but rather the combined action of EBNA-3A's multiple functions involved in cell cycle control and apoptosis frames the phenotype of the EBNA-3A deficient LCLs.

It should be noted, that for some genes controlled by EBNA-3A a plausible function cannot be suggested. In particular, elevated levels of the tumor suppressor and CDK substrate pRB in EBNA-3A proficient LCLs are unexpected and cannot be explained easily.

Other target genes are likely to exert their function *in vivo*. Cadherin 1 (CDH1) is a suppressor of tumor invasion and metastasis, cellular adhesion, antagonist of ß-catenin/Wnt signaling and interferes with growth factor signaling [37]. The chemokine CXCL10 is a chemoattractant for NK and cytotoxic T cells, which is critical for the control of Herpes simplex virus 1 and 2 replication *in vivo* [38],[39]. Matrilysin, MMP7, a matrix metalloprotease which degrades extracellular and non extracellular substrates is found at elevated levels in colon carcinoma and correlates with malignant progression. In addition, MMP7 overexpression has also been reported for a variety of cancers, including those of the oesophagus, stomach, pancreas, lung, colon/rectum and breast cancer, while S100A4 is a mediator of metastasis [40],[and references therein,41]. Thus, genes regulated by EBNA-3A are likely to control the viral life cycle or contribute to the malignancy of the infected B cell *in vivo* in addition to promoting the growth transformation process *in vitro*. Unfortunately, in the absence of a small animal model for EBV infection it is not feasible to evaluate the contribution of these target genes to the pathogenesis of EBV associated diseases.

We consider our study to be a significant contribution to the identification of cellular EBNA-3A target genes, which might include potential therapeutic targets and at the same time provide important tools to study the molecular mechanism of target gene regulation by EBNA-3A.

## Materials and Methods

### Construction of the EBNA-3A negative EBV mutants (EBV-E3AmtA and EBV-E3AmtB)

The targeting vector (bch202) which was used to generate the recombinant B95.8 mutant EBV-E3AmtB was generated by inserting the SmaI-HindIII fragment of pCP15 [42] into a genomic fragment of EBV corresponding to nucleotide position 74948–86110 according to NCBI: AJ 507799.2. The insertion replaced viral sequences corresponding to position 79950–83064, thus deleting the entire ORF of EBNA-3A. The targeting construct (Be694) which was used to generate the recombinant B95.8 mutant EBV-E3AmtA was generated by inserting the SmaI-HindIII derived pCP15 fragment into the BamHI site at position 80418 of the EBV genome. This insertion causes disruption of the EBNA-3A ORF at aminoacid position 126. Both targeting constructs were linearized and introduced into the strain BJ5183, which was pretransformed with p2089, the EBVwt [20],[43]. A map of the genomic sequences is provided in Figure S3A.

### Cell lines

The EBV-negative DG75 Burkitt's lymphoma cell line [44], the EBV-positive Burkitt's lymphoma cell line Raji [45], the EBV-positive cell lines 721 [46], Jijoye and ER/EB2-5 [47],[48], HeLa cells, MRC5 primary human fibroblasts (ATCC) and HEK293 cells [49] were cultivated in RPMI 1640 supplemented with 10% fetal calf serum, 100 U/ml penicillin, 100 µg/ml of streptomycin, and 4 mM glutamine at 37°C in a 6% CO_2_ atmosphere. The growth medium for ER/EB2-5 was supplemented with 1 µM ß-estradiol. In order to maintain standardized cell culture conditions EBVwt, EBV-E3AmtA and EBV-E3AmtB infected B cells were split every third day and re-seeded at a density of 2×10^5^ cells per ml in growth medium containing 20% fetal calf serum. HEK293 cells stably transfected with EBVwt (p2089) or EBVΔE2 (p2491) have been described previously [16],[20]. EBV-E3AmtA or EBV-E3AmtB producing HEK293 cells were generated by lipofection with the respective Maxi-EBV DNA and selected for plasmid maintenance by supplementing the cell culture medium with 100 µg/ml of Hygromycin B.

### Production and quantification of viral supernatants

HEK293 transfectants carrying the recombinant virus plasmid were induced for virus production by cotransfection of 0.5 µg of the plasmids p509 encoding BZLF1 and p2670 encoding BALF4 per one 6-well in 3 ml cell cultures [50]. The supernatants of the transfectants were harvested 3 days p.i. and passed through a 0.8 µm filter. For quantification of viral titers 3×10^5^ Raji cells were infected with serial dilutions of viral supernatants in 1 ml cultures and the percentage of GFP positive cells was determined by FACS analysis 4 days p.i.. The concentration of viral stocks was expressed as the number of green Raji units (GRU). In order to produce high titer virus stocks, virus particles were pelleted by centrifugation at 25,000 rpm in a Beckmann SW28 rotor for 4 hours and then resuspended in 1/7 of the initial volume using cell culture medium.

### Growth transformation of human primary B cells

Human primary B cells were isolated from adenoids, depleted of T cells by rosetting with sheep erytrocytes, and purified by Ficoll-Hypaque density-gradient centrifugation. Successful purification was controlled by FACS analysis. For comparative limiting dilution analysis of growth transformation efficiencies of different viral mutants 1×10^5^ CD19 positive B cells/well were seeded in 96-well cluster plates on lethally irradiated MRC5 feeder layer and infected, using serially diluted normalized virus stocks, with EBVwt, EBV-E3AmtA or EBVΔE2 in a total volume of 200 µl. For each virus dilution groups of 48 wells were plated. Once per week 100 µl of the culture medium was replaced with fresh medium. 5 weeks p.i. the number of wells with proliferating B cells was determined for each virus and degree of dilution and was calculated as the “percentage of positive wells” with respect to the 48 wells/group plated. The average percentage of positive wells per virus dilution was calculated from experiments performed with four different donors and was plotted against the GRUs/well, which had been used for infection. According to the zero term of the Poisson equitation the perpendicular dropped at 63% of positive wells identifies the average number of GRUs necessary to establish one proliferating B cell culture. For the establishment of long-term B cell lines 1–2×10^5^ CD19 positive B cells were infected with 1000 GRUs of EBVwt or different viral mutants and plated on lethally irradiated MRC5 feeder layer in a total volume of 200 µl/well in a 96-well cluster plate. Once per week 100 µl of the culture medium was replaced with fresh medium. 28–35 days p.i. proliferating B cell cultures were removed from feeder layers and expanded indefinitely in suspension cultures.

### Thymidine incorporation assay

CD19 positive B cells were plated at 2, 1 or 0.5×10^5^ cells/well in 96-well cluster plates either on irradiated MRC5 feeder layer or without feeder cells and were infected with 3000 or 4500 GRUs of EBVwt or mutant virus stocks (as individually described in the figure legends) in a total volume of 200 µl. Control cultures were set up with uninfected B cells or feeder cells only. At each investigated point in time 3–6 microcultures were pulsed with 0.5 µCi [^3^H]-thymidine for 16 hours. Subsequently cells were harvested with a Packard FilterMate Harvester on UniFilter-96, GF/C plates and the amount of [^3^H]-thymidine incorporated into DNA was measured in a TopCount Microplate Scintillation Counter.

### LCL Growth curves

LCLs were seeded at an initial density of 2×10^5^ cells/ml in 10 ml cultures. Viable cell counts were determined by trypan blue exclusion (GIBCO) at the indicated points in time, using a hemocytometer and averaging a total of three cell counts per cell line for a given time point. Cultures were subsequently re-seeded at 2×10^5^ cells/ml. Total cell numbers were calculated based on the expansion of the cultures over time.

Conditioned medium was harvested from LCL cultures infected with EBVwt grown to 4–5×10^5^ cells/ml or from feeder cultures at 70% confluency and were passed through a 0.45 µm filter. Since supernatants derived from wt LCL cultures might contain EBVwt virus particles due to the occurrence of spontaneous lytic cells, the respective supernatants were proven to be virtually EBVwt virus free by incubation with Raji cells and subsequent FACS analysis for GFP positive cells.

Experiments using conditioned medium were performed by cultivating 2×10^5^ cells/ml in fresh medium or medium supplemented with 50% conditioned medium derived from either wt LCLs or fibroblast feeder cells. Viable cells were counted and re-seeded at 2×10^5^ cells/ml every third day. Supernatants were prepared freshly each time. For co-cultivation experiments with feeder cells LCLs were seeded at an initial density of 2×10^6^ cells in 10 ml cultures either on 5×10^5^ lethally irradiated MRC5 feeder cells (70% confluent) or without feeder cells. After three days viable cell numbers were determined by trypan blue exclusion and corrected for the fraction of feeder cells.

### Flow-cytometric analysis

Purification of B cells from adenoids was controlled using PE-coupled α-CD19 and FITC-coupled α-CD3 antibodies (Diatec). B cell origin of established LCLs was analyzed using an APC-coupled α-CD19 antibody or an APC-coupled isotype control (BD Biosciences). BrdU-assays (APC BrdU Flow kit; BD Biosciences) and Annexin V-apoptosis assays (Annexin V-Cy5 and 7-AAD; BD Biosciences) were performed according to the manufacturer's protocol. Fluorescence of cells was detected and analyzed using a FACSCalibur system and CellQuest Pro software (BD Biosciences).

### Southern blot and PCR analysis of genomic DNA

Southern blot and PCR analysis was performed as described [51]. Primers used for generation of southern probes were Pr-fw CGC TGA AAT TCG AGT CTT GAG C and Pr-rev GTC AGT ACA CCA TCC AGA GC. Alternatively, genomic DNA was analyzed for the correct state of the EBNA-3A gene locus by PCR using Primers p1 TTG TGC AGG AAC AGG TAT CG, p2 TCC TCC CAG ATT TTC GTG AG, p3 GTC TGT TGT GCC CAG TCA T and p4 GCG GTG TTG GTG AGT CAC AC. Co-infection of LCLs with EBV strain type II was excluded by PCR analysis using Primers EBV-I-fw TTG TGC AGG AAC AGG TAT CG and EBV-I-rev CTA TGG CTC GTG TGT CGA TG specific for EBV strain type I genomic sequences and primers EBV-II-fw GTT CAG CTC CAG CAC AAC AC and EBV-II-rev GGG TGG TCA TTC TCC ATT TG specific for EBV strain type II genomic sequences. Primers GAPDH-fw CGA GAT CCC TCC AAA ATC AA and GAPDH-rev TTC AGC TCA GGG ATG ACC TT were used as a control.

### Western blot analysis

Western blot analysis was performed as described [51] and the following antibodies were used: anti-EBNA-1 (EBNA1-1H4), anti-EBNA-2 (R3-1-3) and anti-EBNA-3A (E3AN4A5) (produced in collaboration with E. Kremmer). The p16 antibody JC8 was a kind gift from Ed Harlow. The antibodies for p14 (Sigma), GAPDH (Chemicon) and polyclonal antibodies against EBNA-3A and EBNA-3C (Ex-alpha Biologicals, Inc) are commercially available.

### Microarray analysis and relative quantification of viral and cellular transcripts by real-time RT-PCR

Microarray analysis starting from 1 µg of total cellular RNA was performed using the HG-U133A 2.0 Affymetrix array according to the manufacturer's protocol. Affymetrix CEL files were processed as described previously [21] and significantly regulated genes were identified by applying the Limma (linear models for microarray analysis) t-test between the 5 wt LCLs and the 9 EBNA-3A negative LCLs [52]. P-values were corrected for multiple testing using the algorithm proposed by Benjamini and Hochberg [53]. Additional filtering based on the fold change between the two conditions was applied with different stringency, individually described in the legend of the tables and figures. Unsupervised hierarchical clustering was performed using *Genesis*, available at http://genome.tugraz.at [54]. Real-time RT-PCR analysis was performed as described previously [21]. Primers used for real-time RT-PCR are summarized in Table S2. Cycling conditions were 1 cycle of 95°C for 10 min and 40 cycles of denaturation (95°C for 1 s), annealing (see Table S2, for 10 s), and extension (72°C for 25 s). PCR products were examined by melting curve analysis and the expected fragment size was verified by agarose gel electrophoresis. To account for differences in reaction efficiencies, a standard curve was generated for each individual primer pair by using serial dilutions of PCR products as templates for amplification and by plotting the crossing points versus the known dilutions. All data were normalized for the relative abundance of the 18S rRNA transcript. The abundance of each target transcript could thus be compared across different RNA samples tested.

### Gene ontology analysis

The *DAVID Functional Annotation Tool*, available at http://david.abcc.ncifcrf.gov (version 2008) was used to calculate over-representation of GO-terms among genes differentially regulated at least 2-fold between wt and EBNA-3A negative LCLs (p≤0.05) [55]. Statistical measures for specific enrichment were assigned by means of an EASE score, which indicates the probability that a given GO-term is more highly enriched among the target set than it would be expected by random chance based on the total number of genes represented on the Affymetrix array HG-U133A 2.0. Functional groups enriched within EBNA-3A target genes were considered as meaningful in case of EASE-scores≤0.01.

### Accession numbers for genes and proteins

Cellular genes or proteins [official gene symbol]

ADAMDEC: 27299; ALOX5: 240; AHR: 196; BAG3: 9531; Bim [BCL1L11]: 10018; BIN1: 274; BIRC3: 330; BMP4: 652; BZW2: 28969; CBF1/RBPJ/CSL: 3516; CCL2: 6347; CCL3: 6348; CD3D: 915; CD3E: 916; CD3G: 917; CD3W: 918; CD3Z [CD247]: 919; CD5: 921; CD19: 930; CD20 [MS4A1]: 931; CD21 [CR2]: 1380; CD23 [FCER2]: 2208; CD40: 958; CD86: 942; CDCP1: 64866; CDH1: 999; CDK5R1: 8851; *c-myc* [MYC]: 4609; CTBP1: 1487; CTBP2: 1488; cyclin D2 [CCND2]: 894; CXCL10: 3627; CXCR4: 7852; FLJ20054 [DENND1B]: 163486; DNAJA1: 3301; ENTH [CLINT]: 9685; FAM13A1: 10144; FAM49A: 81553; MLSTD1 [FAR2]: 55711; FLNA: 2316; GAPDH: 2597; GIMAP5: 55340; HSPA1A: 3303; HSPA1B: 3304; HSPA1L: 3305; HSPA2: 3306; HSPA6: 3310; HSPA7: 3311; HSPCA [HSP90AA1]: 3320; HSPCA [HSP90AA2]: 3324; ITGA4: 3676; ITPR3: 3710; JAG1: 182; LST1: 7940; LTA: 4049; MMP7: 4316; NCALD: 83988; NOL: 8996; p14/ARF [CDKN2A]: 1029; p16/INK4A [CDKN2A]: 1029; p15 [CDKN2B]: 1030; p18 [CDKN2C]: 1031; p19 [CDKN2D]: 1032; p21 [CDKN1A]: 1026; p27 [CDKN1B]: 1027; p57 [CDKN1C]: 1028; PBX3: 5090; PDE4D: 5144; PERP: 64065; PLEKHC1 [FERMT2]: 10979; PU.1 [SPI1]: 6688; RB1: 5925; S100A4: 6275; S100A10: 6281; SPHK1: 8877; STK17B; 9262; STK39: 27347; TCL1A: 8115; TMEM45A: 55076; TTF2: 8458

Viral genes or proteins

BALF4: Q777B0; BZLF1: Q777E5; EBNA-1: Q777E1; EBNA-2: P12978; EBNA-3A: Q8AZJ8; EBNA-3B: Q777E8; EBNA-3C: Q777E7; LMP1: Q777A4; LMP2A: Q777H4; LMP2B: 3783760

## Supporting Information

Figure S1 Irradiated fibroblast feeder layers support cell cycle entry of primary human B cells infected with EBNA-3A deficient viruses under suboptimal culture conditions. Cell cycle entry of primary human B cells after infection with EBVwt, EBV-E3AmtA and EBVΔE2 was analyzed in the absence or presence of fibroblast feeder layer by thymidine incorporation assays. Briefly, B cells were plated on lethally irradiated MRC5 feeder layer or kept without feeder cells at 2, 1 or 0.5×10^5^ B cells per single well of a 96-well plate and were infected with 4500 GRUs of EBVwt, EBV-E3AmtA or EBVΔE2. At day 0, 2, 4 and 7 p.i. cells were pulsed with [^3^H]-thymidine and analyzed for thymidine incorporation. Results are given as mean values±standard deviation derived from triplicates for each indicated point in time and experimental condition.
(1.25 MB TIF)

Figure S2 Verification of the B cell origin of EBNA-3A negative LCLs. (A) Example for a typical B cell purification from human adenoids by T cell rosetting. Prior to infection with EBV wt or mutant viral stocks, primary human B cells were routinely purified from adenoids of small children (aged below 4 years) by rosetting of T cells with sheep erythrocytes. Successful purification was monitored by FACS analysis after staining of cells with PE-coupled α-CD19 and FITC-coupled α-CD3 antibodies. Typically, the whole cell population prior to purification contained 22–25% T cells and 72–75% B cells. By T cell rosetting, the T cell fraction was in general reduced to 0.2–1.5% while the B cell fraction was enriched to 96–97%. (B) LCLs established by infection of purified B cells with EBVwt or either EBV-E3AmtB (D2, D3) or EBV-E3AmtA (D1, D4–D7) viral stocks were proven for B cell origin by FACS analysis after staining of cells with APC-coupled α-CD19 antibody. Cells stained with an APC-coupled isotype control were used as negative control.
(2.70 MB TIF)

Figure S3 Southern blot analysis of the recombinant Maxi-EBV mutants. (A) Section of the restriction map of the EBVwt genome and the two distinct recombinant viral mutants generated for this study. Restriction sites for *Sph*I digestion and expected fragment sizes, hybridizing to the indicated genomic probe, are depicted. (B) HEK293 cells stably transfected with EBVwt, EBV-E3AmtA or EBV-E3AmtB were analyzed for the correct state of the modified EBNA-3A gene locus by southern blot analysis prior to production of viral supernatants. For EBV-E3AmtA several clones (cl.) of HEK293 transfectants were tested. (C) Southern blot hybridization proved the correct state of the EBNA-3A gene locus in established wt and EBNA-3A negative LCLs derived from three different donors (D1–D3). The corresponding HEK293 virus producing helper cell lines were included as a control.
(1.56 MB TIF)

Figure S4 EBNA-3A negative LCLs are not co-infected with EBV strain type II. Wt and EBNA-3A negative LCLs derived from 7 individual donors (D1–D7) were analyzed for co-infection with EBV type II by PCR analysis of total cellular DNA. The position of primers for both EBV strains is located within exon 1 and exon 2 of the EBNA-3A gene locus (A). Primers specific for EBV strain type I yielded a PCR product of 379 bp. No PCR product is generated with DNA originating from EBV-E3AmtB LCLs with the whole EBNA-3A gene locus deleted (B). Primers specific for EBV strain type II produced a 364 bp product. The EBV type II infected cell line ER/EB2-5 was used as a control (C). To control for successful DNA preparation PCR analysis was carried out using primers for the GAPDH gene locus (D).
(2.17 MB TIF)

Figure S5 The reduced proliferation rates of EBNA-3A negative LCLs can neither be corrected by cell culture supernatants derived from wt LCLs or fibroblast feeder cells, nor by direct cultivation on irradiated fibroblast feeder layers. (A) EBNA-3A negative LCLs derived from two individual donors by infection of B cells with EBV-E3AmtA (D8, right panel) or EBV-E3AmtB (D3, left panel) were resuspended at an initial density of 2×10^5^ cells per ml in either fresh medium or medium supplemented with 50% of cell culture supernatant (SN) derived from the respective wt LCL or from MRC5 fibroblast feeder cells. Since supernatants derived from wt LCL cultures might contain EBVwt virus particles due to the occurrence of spontaneous lytic cells, the respective supernatants were proven to be virtually EBVwt virus free as shown in (B). Viable cell counts were determined every third day and cells were re-seeded at 2×10^5^ cells per ml, again using either fresh medium or medium supplemented with 50% of the respective cell culture supernatant. For comparison, the corresponding wt LCLs grown in fresh medium were included for each donor. Results are given as total numbers of viable cells corrected for the expansion of the cultures over time. The data are shown as mean values of triplicates. (B) Cell culture supernatants obtained from wt LCLs were tested for EBVwt virus load by FACS analysis. Briefly, 3×10^5^ Raji cells were incubated with 0.5 ml of wt LCL derived cell culture supernatant in a 1 ml culture for 4 days and were analyzed for GFP-positive cells by FACS analysis (left panel). As a positive control Raji cells were incubated with 0.5 ml of supernatants derived from the EBVwt HEK293 producer cell line after induction of EBV's lytic cyle (middle panel). Untreated Raji cells were used as negative control (right panel). The results are given as percentage of GFP positive cells in the lower right quadrant. (C) Wt and EBNA-3A negative LCLs analyzed in (A) were seeded at an initial density of 2×10^5^ cells per ml either on irradiated MRC5 feeder layers or without (w/o) feeder cells. Viable cell numbers were determined after three days. Results are given as mean values±standard deviation derived from triplicates.
(0.94 MB TIF)

Figure S6 Expression profiles of wt and EBNA-3A negative LCLs. Shown are 380 probe sets displaying at least 2-fold changes in expression levels with significance p≤0.05 in EBNA-3A negative LCLs compared to wt LCLs. Vertical columns represent data obtained for each individual cell line by hybridization to a single microarray, while horizontal rows represent data obtained for a particular probe set across all cell lines. After normalization of expression values on a scale ranging from −2.0 to 2.0 for each probe set, an unsupervised hierarchical clustering analysis was performed, using Pearson correlation as a measure for similarity between genes and complete linkage as a clustering allocation algorithm. High expression values are represented by red, low expression values by green and medium values by black.
(1.63 MB PDF)

Table S1 Compilation of EBNA-3A regulated cellular genes combined with a list of selected EBNA-2 target genes, which are either co- or counter-regulated. Expression levels of 380 out of 22,277 probe sets were found to be at least 2-fold different (p≤0.05) between wt and EBNA-3A-negative LCLs, corresponding to 296 genes and 3 not yet annotated loci. Of these 296 genes, 129 genes were found to be down-regulated in wt LCLs, while 167 genes were found to be up-regulated. For each gene the table contains fold-changes and p-values for the probe set, which showed the strongest regulation (designated in bold). For genes that were identified more than once, the additional probe sets are also listed. To assess the overlap with EBNA-2 cellular target genes, probe sets were mapped to gene symbols (including aliases) and compared to EBNA-2 targets identified by us and others. EBNA-3A target genes that were already described as a target of EBNA-2 by Zhao and colleagues or by Spender and colleagues are indicated. For overlapping EBNA-2 target genes identified by us, fold-changes and p-values are displayed, originating from our former expression profiles. The analysis highlighted an overlap of 75 genes, corresponding to 25.3% of EBNA-3A target genes also to be regulated by EBNA-2.
(3.28 MB XLS)

Table S2 Primers used for real-time RT-PCR and annealing temperatures.
(0.09 MB DOC)

## References

[1] Tomkinson B, Robertson E, Kieff E (1993). Epstein-Barr virus nuclear proteins EBNA-3A and EBNA-3C are essential for B-lymphocyte growth transformation.. J Virol.

[2] Maruo S, Johannsen E, Illanes D, Cooper A, Kieff E (2003). Epstein-Barr Virus nuclear protein EBNA3A is critical for maintaining lymphoblastoid cell line growth.. J Virol.

[3] Lee W, Hwang YH, Lee SK, Subramanian C, Robertson ES (2001). An Epstein-Barr virus isolated from a lymphoblastoid cell line has a 16-kilobase-pair deletion which includes gp350 and the Epstein-Barr virus nuclear antigen 3A.. J Virol.

[4] Kempkes B, Pich D, Zeidler R, Sugden B, Hammerschmidt W (1995). Immortalization of human B lymphocytes by a plasmid containing 71 kilobase pairs of Epstein-Barr virus DNA.. J Virol.

[5] Bain M, Watson RJ, Farrell PJ, Allday MJ (1996). Epstein-Barr virus nuclear antigen 3C is a powerful repressor of transcription when tethered to DNA.. J Virol.

[6] Bourillot PY, Waltzer L, Sergeant A, Manet E (1998). Transcriptional repression by the Epstein-Barr virus EBNA3A protein tethered to DNA does not require RBP-Jkappa.. J Gen Virol.

[7] Cludts I, Farrell PJ (1998). Multiple functions within the Epstein-Barr virus EBNA-3A protein.. J Virol.

[8] Hickabottom M, Parker GA, Freemont P, Crook T, Allday MJ (2002). Two nonconsensus sites in the Epstein-Barr virus oncoprotein EBNA3A cooperate to bind the co-repressor carboxyl-terminal-binding protein (CtBP).. J Biol Chem.

[9] Le Roux A, Kerdiles B, Walls D, Dedieu JF, Perricaudet M (1994). The Epstein-Barr virus determined nuclear antigens EBNA-3A, -3B, and -3C repress EBNA-2-mediated transactivation of the viral terminal protein 1 gene promoter.. Virology.

[10] Marshall D, Sample C (1995). Epstein-Barr virus nuclear antigen 3C is a transcriptional regulator.. J Virol.

[11] Waltzer L, Perricaudet M, Sergeant A, Manet E (1996). Epstein-Barr virus EBNA3A and EBNA3C proteins both repress RBP-J kappa-EBNA2-activated transcription by inhibiting the binding of RBP-J kappa to DNA.. J Virol.

[12] Radkov SA, Bain M, Farrell PJ, West M, Rowe M (1997). Epstein-Barr virus EBNA3C represses Cp, the major promoter for EBNA expression, but has no effect on the promoter of the cell gene CD21.. J Virol.

[13] Dalbies-Tran R, Stigger-Rosser E, Dotson T, Sample CE (2001). Amino acids of Epstein-Barr virus nuclear antigen 3A essential for repression of Jkappa-mediated transcription and their evolutionary conservation.. J Virol.

[14] Maruo S, Johannsen E, Illanes D, Cooper A, Zhao B (2005). Epstein-Barr virus nuclear protein 3A domains essential for growth of lymphoblasts: transcriptional regulation through RBP-Jkappa/CBF1 is critical.. J Virol.

[15] Cooper A, Johannsen E, Maruo S, Cahir-McFarland E, Illanes D (2003). EBNA3A association with RBP-Jkappa down-regulates c-myc and Epstein-Barr virus-transformed lymphoblast growth.. J Virol.

[16] Kelly GL, Milner AE, Tierney RJ, Croom-Carter DS, Altmann M (2005). Epstein-Barr virus nuclear antigen 2 (EBNA2) gene deletion is consistently linked with EBNA3A, -3B, and -3C expression in Burkitt's lymphoma cells and with increased resistance to apoptosis.. J Virol.

[17] Anderton E, Yee J, Smith P, Crook T, White RE (2008). Two Epstein-Barr virus (EBV) oncoproteins cooperate to repress expression of the proapoptotic tumour-suppressor Bim: clues to the pathogenesis of Burkitt's lymphoma.. Oncogene.

[18] Young P, Anderton E, Paschos K, White R, Allday MJ (2008). Epstein-Barr virus nuclear antigen (EBNA) 3A induces the expression of and interacts with a subset of chaperones and co-chaperones.. J Gen Virol.

[19] Kashuba EV, Gradin K, Isaguliants M, Szekely L, Poellinger L (2006). Regulation of transactivation function of the aryl hydrocarbon receptor by the Epstein-Barr virus-encoded EBNA-3 protein.. J Biol Chem.

[20] Delecluse HJ, Hilsendegen T, Pich D, Zeidler R, Hammerschmidt W (1998). Propagation and recovery of intact, infectious Epstein-Barr virus from prokaryotic to human cells.. Proc Natl Acad Sci U S A.

[21] Maier S, Staffler G, Hartmann A, Hock J, Henning K (2006). Cellular target genes of epstein-barr virus nuclear antigen 2.. J Virol.

[22] Zhao B, Maruo S, Cooper A, Chase MR, Johannsen E (2006). RNAs induced by Epstein-Barr virus nuclear antigen 2 in lymphoblastoid cell lines.. Proc Natl Acad Sci U S A.

[23] Spender LC, Lucchesi W, Bodelon G, Bilancio A, Karstegl CE (2006). Cell target genes of Epstein-Barr virus transcription factor EBNA-2: induction of the p55alpha regulatory subunit of PI3-kinase and its role in survival of EREB2.5 cells.. J Gen Virol.

[24] Foo RS, Nam YJ, Ostreicher MJ, Metzl MD, Whelan RS (2007). Regulation of p53 tetramerization and nuclear export by ARC.. Proc Natl Acad Sci U S A.

[25] Srinivasula SM, Ashwell JD (2008). IAPs: what's in a name?. Mol Cell.

[26] Attardi LD, Reczek EE, Cosmas C, Demicco EG, McCurrach ME (2000). PERP, an apoptosis-associated target of p53, is a novel member of the PMP-22/gas3 family.. Genes Dev.

[27] Kogel D, Prehn JH, Scheidtmann KH (2001). The DAP kinase family of pro-apoptotic proteins: novel players in the apoptotic game.. Bioessays.

[28] Maruo S, Wu Y, Ishikawa S, Kanda T, Iwakiri D (2006). Epstein-Barr virus nuclear protein EBNA3C is required for cell cycle progression and growth maintenance of lymphoblastoid cells.. Proc Natl Acad Sci U S A.

[29] Gottschalk S, Ng CY, Perez M, Smith CA, Sample C (2001). An Epstein-Barr virus deletion mutant associated with fatal lymphoproliferative disease unresponsive to therapy with virus-specific CTLs.. Blood.

[30] Chen A, Zhao B, Kieff E, Aster JC, Wang F (2006). EBNA-3B- and EBNA-3C-regulated cellular genes in Epstein-Barr virus-immortalized lymphoblastoid cell lines.. J Virol.

[31] Zhao B, Marshall DR, Sample CE (1996). A conserved domain of the Epstein-Barr virus nuclear antigens 3A and 3C binds to a discrete domain of Jkappa.. J Virol.

[32] Johannsen E, Miller CL, Grossman SR, Kieff E (1996). EBNA-2 and EBNA-3C extensively and mutually exclusively associate with RBPJkappa in Epstein-Barr virus-transformed B lymphocytes.. J Virol.

[33] Allday MJ, Farrell PJ (1994). Epstein-Barr virus nuclear antigen EBNA3C/6 expression maintains the level of latent membrane protein 1 in G1-arrested cells.. J Virol.

[34] Alazard N, Gruffat H, Hiriart E, Sergeant A, Manet E (2003). Differential hyperacetylation of histones H3 and H4 upon promoter-specific recruitment of EBNA2 in Epstein-Barr virus chromatin.. J Virol.

[35] Jimenez-Ramirez C, Brooks AJ, Forshell LP, Yakimchuk K, Zhao B (2006). Epstein-Barr virus EBNA-3C is targeted to and regulates expression from the bidirectional LMP-1/2B promoter.. J Virol.

[36] Zhao B, Sample CE (2000). Epstein-barr virus nuclear antigen 3C activates the latent membrane protein 1 promoter in the presence of Epstein-Barr virus nuclear antigen 2 through sequences encompassing an spi-1/Spi-B binding site.. J Virol.

[37] Jeanes A, Gottardi CJ, Yap AS (2008). Cadherins and cancer: how does cadherin dysfunction promote tumor progression?. Oncogene.

[38] Wuest TR, Carr DJ (2008). Dysregulation of CXCR3 signaling due to CXCL10 deficiency impairs the antiviral response to herpes simplex virus 1 infection.. J Immunol.

[39] Thapa M, Welner RS, Pelayo R, Carr DJ (2008). CXCL9 and CXCL10 expression are critical for control of genital herpes simplex virus type 2 infection through mobilization of HSV-specific CTL and NK cells to the nervous system.. J Immunol.

[40] Beeghly-Fadiel A, Long JR, Gao YT, Li C, Qu S (2008). Common MMP-7 polymorphisms and breast cancer susceptibility: a multistage study of association and functionality.. Cancer Res.

[41] Garrett SC, Varney KM, Weber DJ, Bresnick AR (2006). S100A4, a mediator of metastasis.. J Biol Chem.

[42] Cherepanov PP, Wackernagel W (1995). Gene disruption in Escherichia coli: TcR and KmR cassettes with the option of Flp-catalyzed excision of the antibiotic-resistance determinant.. Gene.

[43] Delecluse HJ, Pich D, Hilsendegen T, Baum C, Hammerschmidt W (1999). A first-generation packaging cell line for Epstein-Barr virus-derived vectors.. Proc Natl Acad Sci U S A.

[44] Ben-Bassat H, Goldblum N, Mitrani S, Goldblum T, Yoffey JM (1977). Establishment in continuous culture of a new type of lymphocyte from a “Burkitt like” malignant lymphoma (line D.G.-75).. Int J Cancer.

[45] Pulvertaft JV (1964). Cytology of Burkitt's Tumour (African Lymphoma).. Lancet.

[46] Kavathas P, Bach FH, DeMars R (1980). Gamma ray-induced loss of expression of HLA and glyoxalase I alleles in lymphoblastoid cells.. Proc Natl Acad Sci U S A.

[47] Kohn G, Mellman WJ, Moorhead PS, Loftus J, Henle G (1967). Involvement of C group chromosomes in five Burkitt lymphoma cell lines.. J Natl Cancer Inst.

[48] Kempkes B, Spitkovsky D, Jansen-Durr P, Ellwart JW, Kremmer E (1995). B-cell proliferation and induction of early G1-regulating proteins by Epstein-Barr virus mutants conditional for EBNA2.. Embo J.

[49] Graham FL, Smiley J, Russell WC, Nairn R (1977). Characteristics of a human cell line transformed by DNA from human adenovirus type 5.. J Gen Virol.

[50] Neuhierl B, Feederle R, Hammerschmidt W, Delecluse HJ (2002). Glycoprotein gp110 of Epstein-Barr virus determines viral tropism and efficiency of infection.. Proc Natl Acad Sci U S A.

[51] Maier S, Santak M, Mantik A, Grabusic K, Kremmer E (2005). A somatic knockout of CBF1 in a human B-cell line reveals that induction of CD21 and CCR7 by EBNA-2 is strictly CBF1 dependent and that downregulation of immunoglobulin M is partially CBF1 independent.. J Virol.

[52] Smyth GK, Michaud J, Scott HS (2005). Use of within-array replicate spots for assessing differential expression in microarray experiments.. Bioinformatics.

[53] Benjamini Y, Hochberg Y (1995). Controlling the false discovery rate: a practical and powerful approach to multiple testing.. Journal of the Royal Statistical Society Series.

[54] Sturn A, Quackenbush J, Trajanoski Z (2002). Genesis: cluster analysis of microarray data.. Bioinformatics.

[55] Dennis G, Sherman BT, Hosack DA, Yang J, Gao W (2003). DAVID: Database for Annotation, Visualization, and Integrated Discovery.. Genome Biol.

