# Supplementary figures and images for: Differential Gene Expression Patterns of EBV Infected EBNA-3A Positive and Negative Human B Lymphocytes
Source: PLoS Pathog. 2009 Jul 3;5(7):e1000506. doi: 10.1371/journal.ppat.1000506 (PMC2700271; doi:10.1371/journal.ppat.1000506)

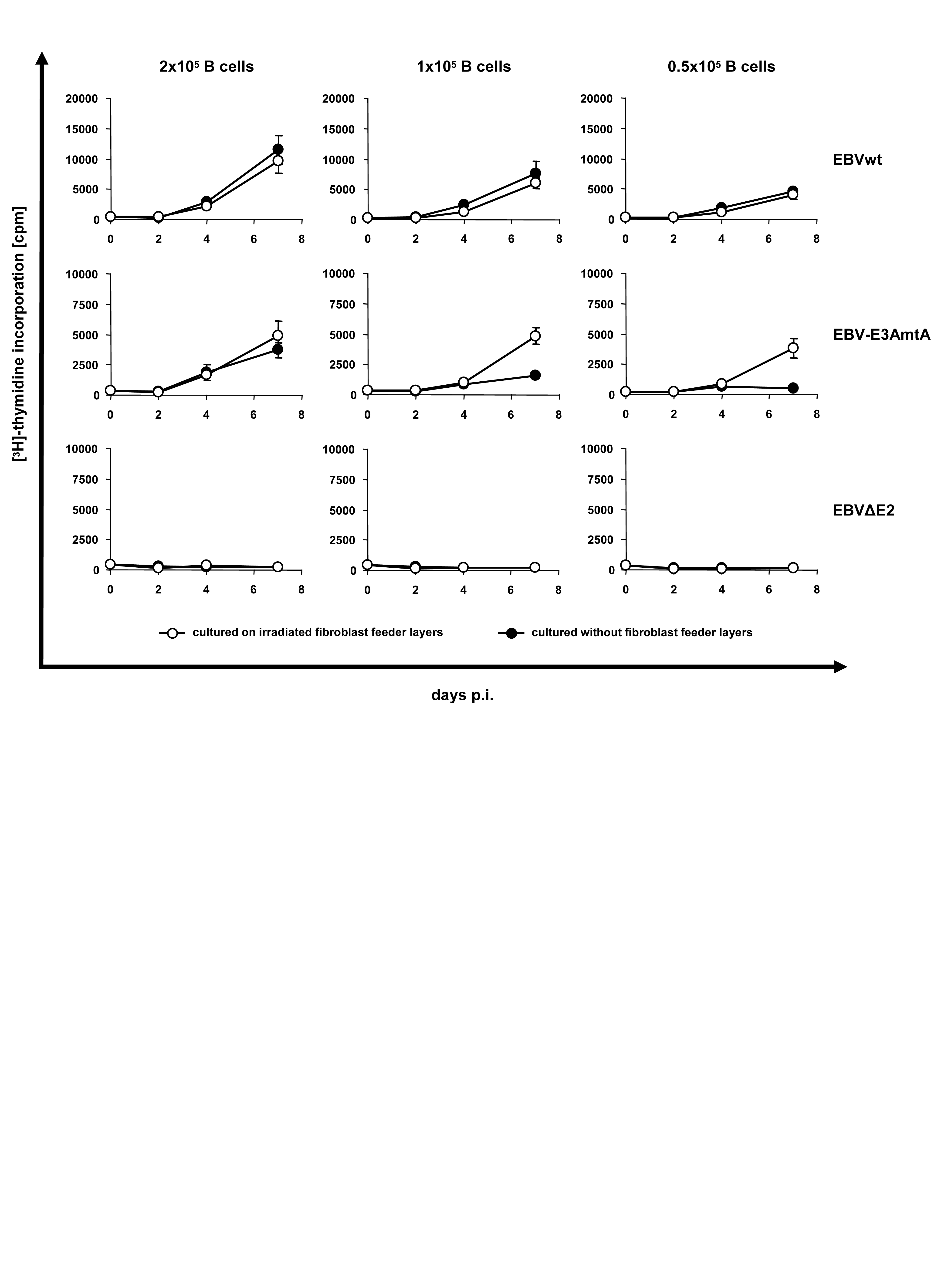

Supplement: Figure S1 — Irradiated fibroblast feeder layers support cell cycle entry of primary human B cells infected with EBNA-3A deficient viruses under suboptimal culture conditions. Cell cycle entry of primary human B cells after infection with EBVwt, EBV-E3AmtA and EBVΔE2 was analyzed in the absence or presence of fibroblast feeder layer by thymidine incorporation assays. Briefly, B cells were plated on lethally irradiated MRC5 feeder layer or kept without feeder cells at 2, 1 or 0.5×10^5^ B cells per single well of a 96-well plate and were infected with 4500 GRUs of EBVwt, EBV-E3AmtA or EBVΔE2. At day 0, 2, 4 and 7 p.i. cells were pulsed with [3H]-thymidine and analyzed for thymidine incorporation. Results are given as mean values±standard deviation derived from triplicates for each indicated point in time and experimental condition. (1.25 MB TIF) [file ppat.1000506.s001.tif]

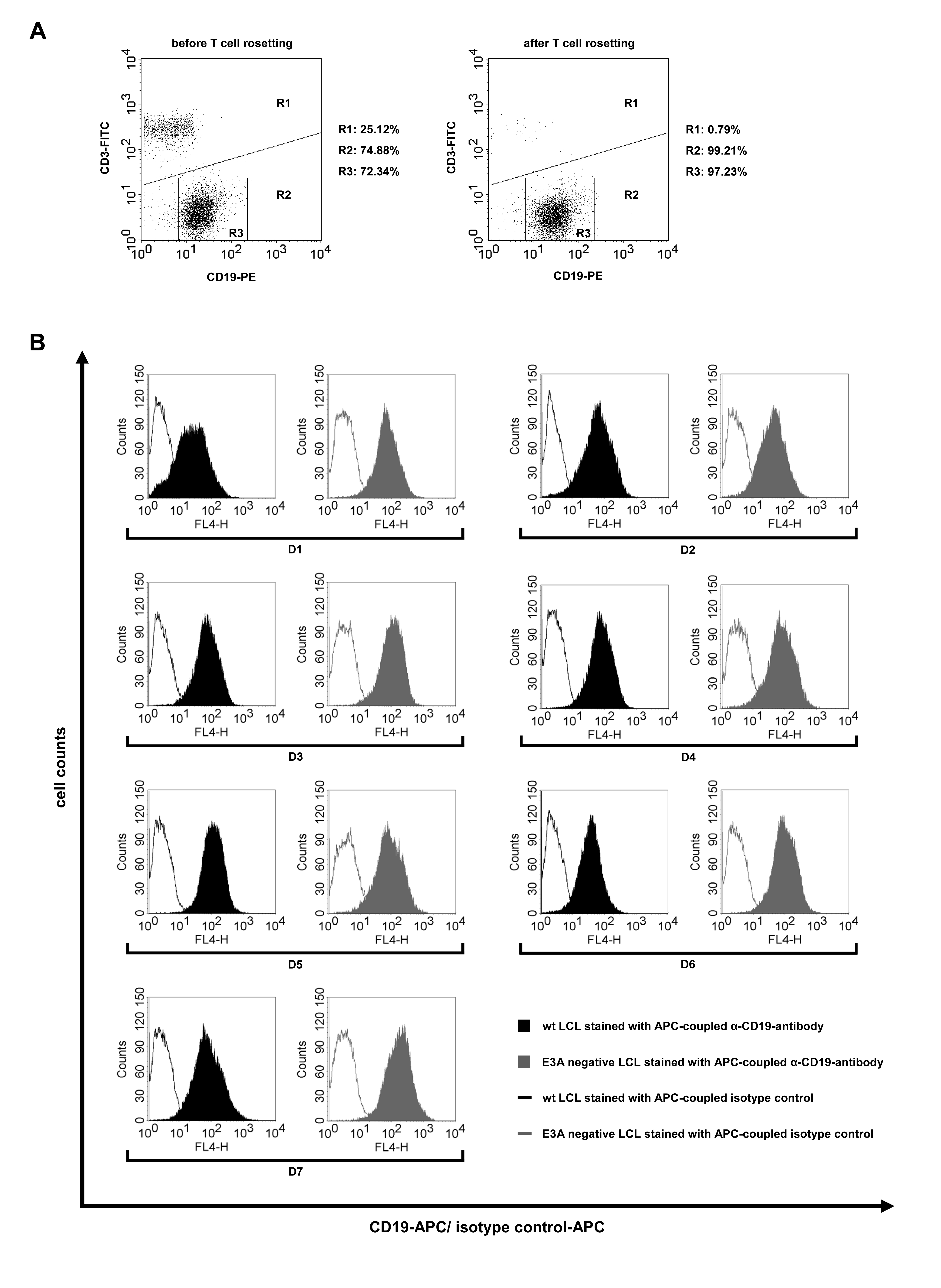

Supplement: Figure S2 — Verification of the B cell origin of EBNA-3A negative LCLs. (A) Example for a typical B cell purification from human adenoids by T cell rosetting. Prior to infection with EBV wt or mutant viral stocks, primary human B cells were routinely purified from adenoids of small children (aged below 4 years) by rosetting of T cells with sheep erythrocytes. Successful purification was monitored by FACS analysis after staining of cells with PE-coupled α-CD19 and FITC-coupled α-CD3 antibodies. Typically, the whole cell population prior to purification contained 22–25% T cells and 72–75% B cells. By T cell rosetting, the T cell fraction was in general reduced to 0.2–1.5% while the B cell fraction was enriched to 96–97%. (B) LCLs established by infection of purified B cells with EBVwt or either EBV-E3AmtB (D2, D3) or EBV-E3AmtA (D1, D4–D7) viral stocks were proven for B cell origin by FACS analysis after staining of cells with APC-coupled α-CD19 antibody. Cells stained with an APC-coupled isotype control were used as negative control. (2.70 MB TIF) [file ppat.1000506.s002.tif]

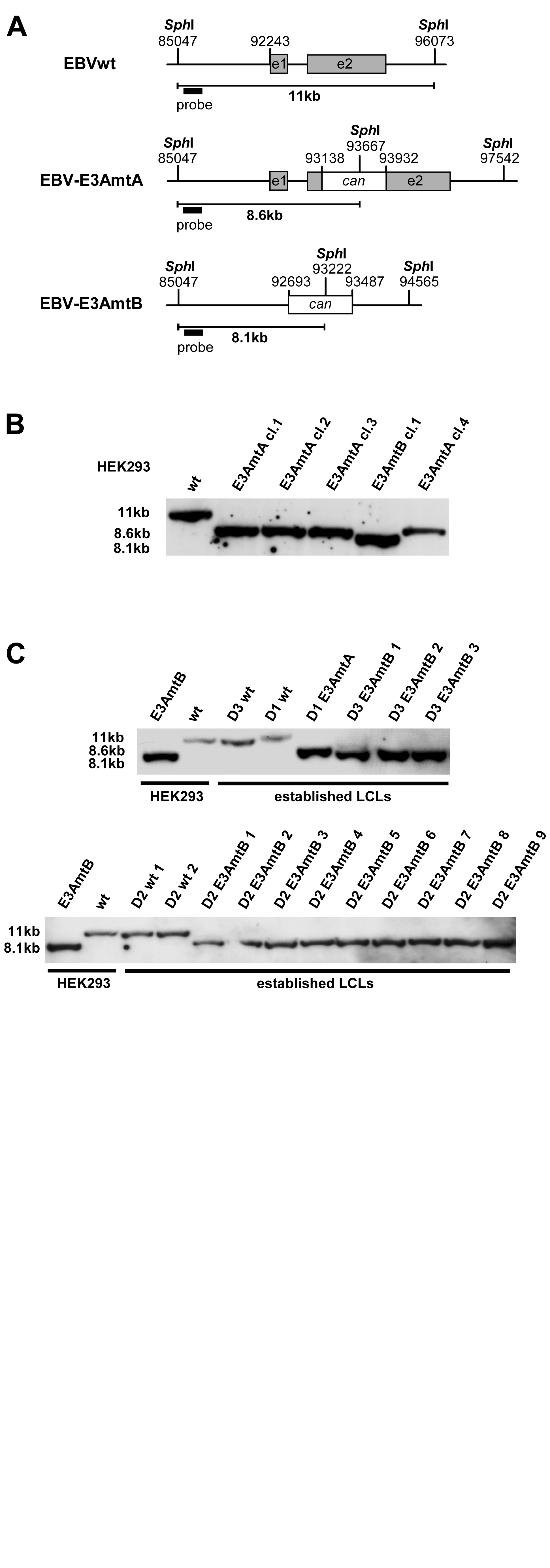

Supplement: Figure S3 — Southern blot analysis of the recombinant Maxi-EBV mutants. (A) Section of the restriction map of the EBVwt genome and the two distinct recombinant viral mutants generated for this study. Restriction sites for SphI digestion and expected fragment sizes, hybridizing to the indicated genomic probe, are depicted. (B) HEK293 cells stably transfected with EBVwt, EBV-E3AmtA or EBV-E3AmtB were analyzed for the correct state of the modified EBNA-3A gene locus by southern blot analysis prior to production of viral supernatants. For EBV-E3AmtA several clones (cl.) of HEK293 transfectants were tested. (C) Southern blot hybridization proved the correct state of the EBNA-3A gene locus in established wt and EBNA-3A negative LCLs derived from three different donors (D1–D3). The corresponding HEK293 virus producing helper cell lines were included as a control. (1.56 MB TIF) [file ppat.1000506.s003.tif]

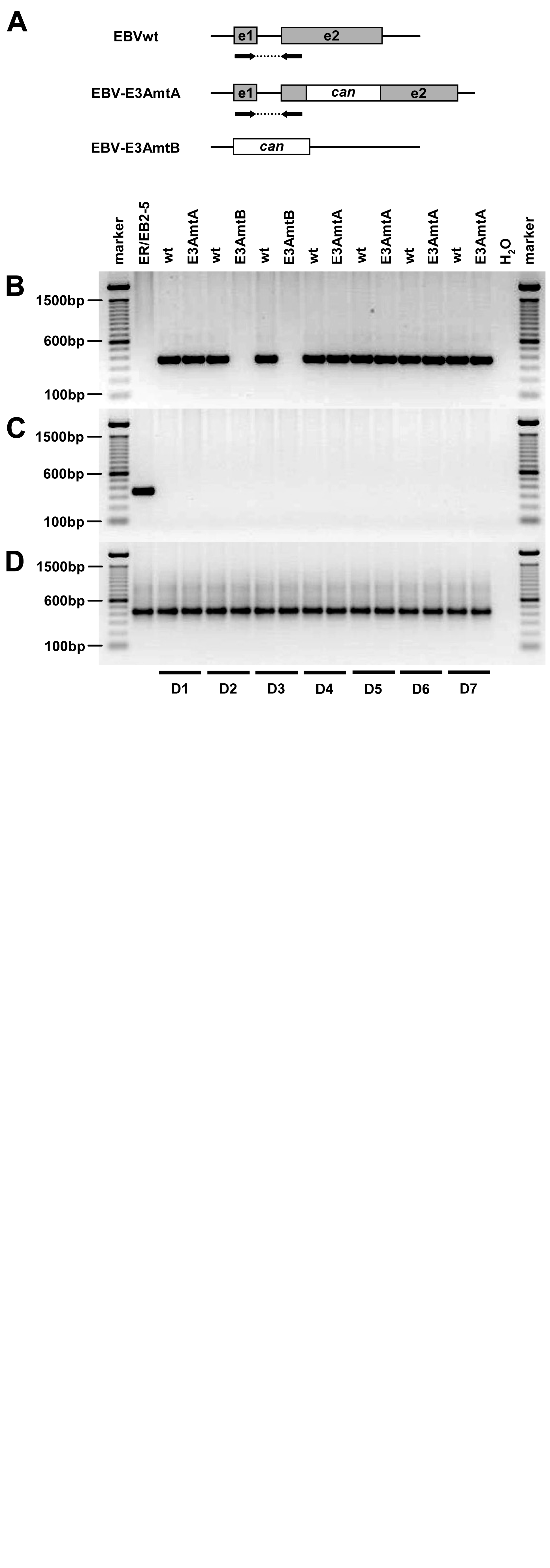

Supplement: Figure S4 — EBNA-3A negative LCLs are not co-infected with EBV strain type II. Wt and EBNA-3A negative LCLs derived from 7 individual donors (D1–D7) were analyzed for co-infection with EBV type II by PCR analysis of total cellular DNA. The position of primers for both EBV strains is located within exon 1 and exon 2 of the EBNA-3A gene locus (A). Primers specific for EBV strain type I yielded a PCR product of 379 bp. No PCR product is generated with DNA originating from EBV-E3AmtB LCLs with the whole EBNA-3A gene locus deleted (B). Primers specific for EBV strain type II produced a 364 bp product. The EBV type II infected cell line ER/EB2-5 was used as a control (C). To control for successful DNA preparation PCR analysis was carried out using primers for the GAPDH gene locus (D). (2.17 MB TIF) [file ppat.1000506.s004.tif]

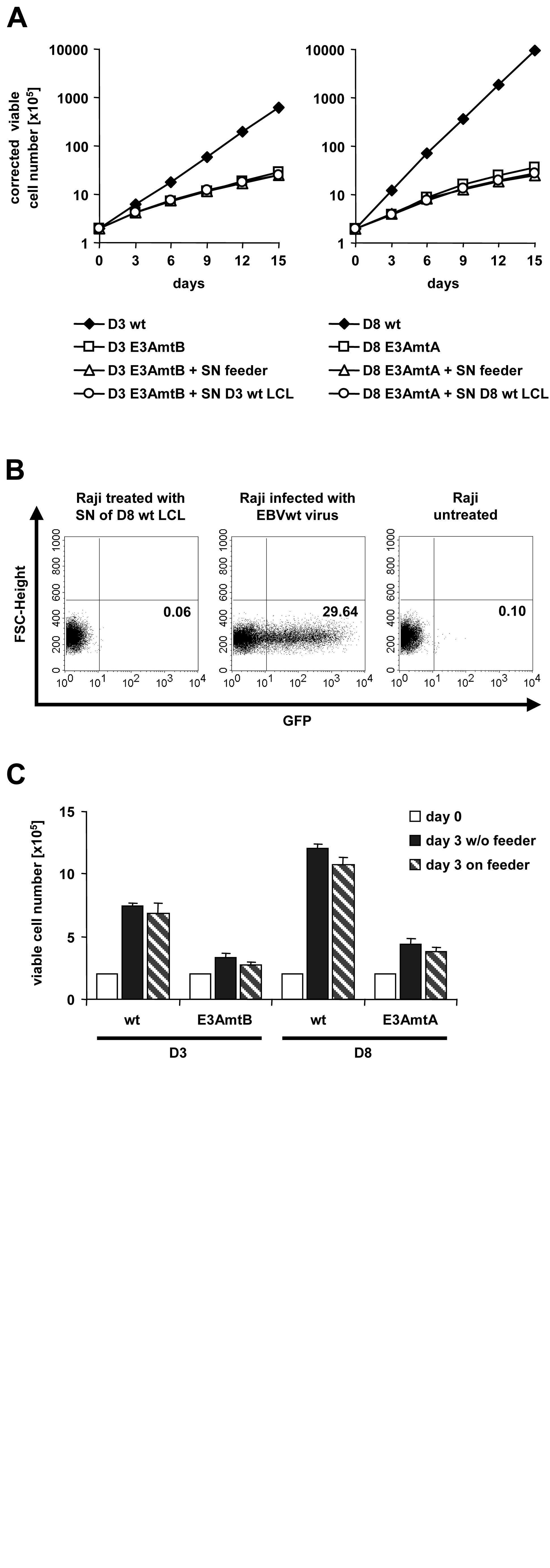

Supplement: Figure S5 — The reduced proliferation rates of EBNA-3A negative LCLs can neither be corrected by cell culture supernatants derived from wt LCLs or fibroblast feeder cells, nor by direct cultivation on irradiated fibroblast feeder layers. (A) EBNA-3A negative LCLs derived from two individual donors by infection of B cells with EBV-E3AmtA (D8, right panel) or EBV-E3AmtB (D3, left panel) were resuspended at an initial density of 2×10^5^ cells per ml in either fresh medium or medium supplemented with 50% of cell culture supernatant (SN) derived from the respective wt LCL or from MRC5 fibroblast feeder cells. Since supernatants derived from wt LCL cultures might contain EBVwt virus particles due to the occurrence of spontaneous lytic cells, the respective supernatants were proven to be virtually EBVwt virus free as shown in (B). Viable cell counts were determined every third day and cells were re-seeded at 2×10^5^ cells per ml, again using either fresh medium or medium supplemented with 50% of the respective cell culture supernatant. For comparison, the corresponding wt LCLs grown in fresh medium were included for each donor. Results are given as total numbers of viable cells corrected for the expansion of the cultures over time. The data are shown as mean values of triplicates. (B) Cell culture supernatants obtained from wt LCLs were tested for EBVwt virus load by FACS analysis. Briefly, 3×10^5^ Raji cells were incubated with 0.5 ml of wt LCL derived cell culture supernatant in a 1 ml culture for 4 days and were analyzed for GFP-positive cells by FACS analysis (left panel). As a positive control Raji cells were incubated with 0.5 ml of supernatants derived from the EBVwt HEK293 producer cell line after induction of EBV's lytic cyle (middle panel). Untreated Raji cells were used as negative control (right panel). The results are given as percentage of GFP positive cells in the lower right quadrant. (C) Wt and EBNA-3A negative LCLs analyzed in (A) were seeded at an initial density of 2×10^5^ [file ppat.1000506.s005.tif]

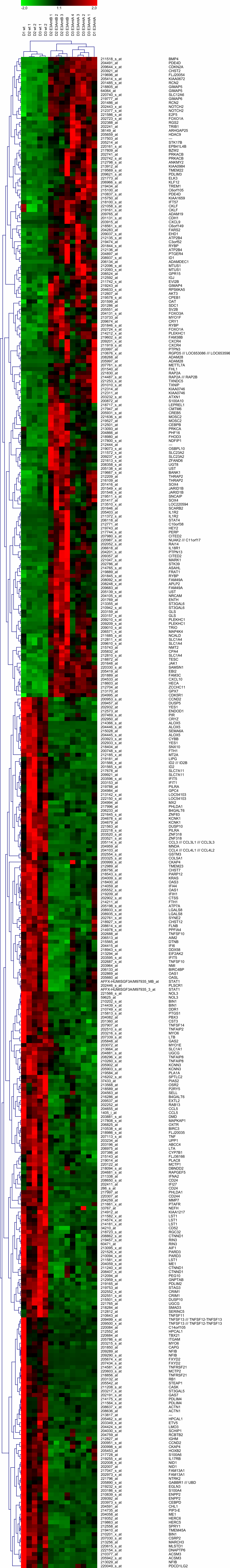

Supplement: Figure S6 — Expression profiles of wt and EBNA-3A negative LCLs. Shown are 380 probe sets displaying at least 2-fold changes in expression levels with significance p≤0.05 in EBNA-3A negative LCLs compared to wt LCLs. Vertical columns represent data obtained for each individual cell line by hybridization to a single microarray, while horizontal rows represent data obtained for a particular probe set across all cell lines. After normalization of expression values on a scale ranging from −2.0 to 2.0 for each probe set, an unsupervised hierarchical clustering analysis was performed, using Pearson correlation as a measure for similarity between genes and complete linkage as a clustering allocation algorithm. High expression values are represented by red, low expression values by green and medium values by black. (1.63 MB PDF) [file ppat.1000506.s006.pdf]
