# Supplementary material for: Differential Gene Expression Patterns of EBV Infected EBNA-3A Positive and Negative Human B Lymphocytes
Source: PLoS Pathog. 2009 Jul 3;5(7):e1000506. doi: 10.1371/journal.ppat.1000506 (PMC2700271; doi:10.1371/journal.ppat.1000506)
Supplement: Table S2 — Primers used for real-time RT-PCR and annealing temperatures. (0.09 MB DOC) [file ppat.1000506.s008.doc]

**Table S2: Primers used for real-time RT-PCR and annealing temperatures**

| **Primer** | **Sequence (5´-3´)** | **Annealing temperature (°C)** |
| --- | --- | --- |
| ADAMDEC1-fw | GAAGAGCACTGACGGGAAAC | 62 |
| ADAMDEC1-rev | ACCAAGGCCACTTGAACATC |
| ALOX5-fw | TCATCGTGGACTTTGAGCTG | 62 |
| ALOX5-rev | AGAAGGTGGGTGATGGTCTG |
| BIN1-fw | GGCTTCCAAGAAGCTGAATG | 60 |
| BIN1-rev | GGTGGTAATCCATCCACAGC |
| BMP4-fw | TCCACAGCACTGGTCTTGAG | 62 |
| BMP4-rev | CGTGTCACATTGTGGTGGAC |
| BZW2-fw | TAAGCATCAGAAGCCAGTGC | 62 |
| BZW2-rev | ATCATCTATGCGCGTTCCTC |
| CCL3-fw | ATGCAGGTCTCCACTGCTG | 60 |
| CCL3-rev | TTTCTGGACCCACTCCTCAC |
| CDH1-fw | GGATGTGCTGGATGTGAATG | 60 |
| CDH1-rev | TTAGGGCTGTGTACGTGCTG |
| C promoter-fw | AGATCAGATGGCATAGAGAC | 63 |
| C promoter-rev | GACCGGTGCCTTCTTAGGAG |
| CXCL10-fw | TGACTCTAAGTGGCATTCAAGG | 60 |
| CXCL10-rev | CCTTTCCTTGCTAACTGCTTTC |
| FAM13A1-fw | GCTTGAAGGCATGAAGGAAC | 62 |
| FAM13A1-rev | CTGTGGGCCTGAATAGATCC |
| FAM49A-fw | GACTGCCTCAGCACAATGAC | 60 |
| FAM49A-rev | TGAAGCATTGCTCGAATCTG |
| FLJ20054-fw | CTCCCAACAATACCCGAGAG | 60 |
| FLJ20054-rev | TCCAATCAGGTATGGCATTG |
| GIMAP5-fw | GAATGGGAGGATTCCAGAGG | 62 |
| GIMAP5-rev | TCAGCTTGGACTCAAACACG |
| LMP-1-fw | GGTGTTCATCACTGTGTCGTTGTC | 66 |
| LMP-1-rev | GCTACTGTTTTGGCTGTACATCGT |
| LMP2A-fw | ATGACTCATCTCAACACATA | 62 |
| LMP2A/B-rev | CATGTTAGGCAAATTGCAAA |
| LMP2B-fw | CAGTGTAATCTGCACAAAGA | 60 |
| LMP2A/B-rev | CATGTTAGGCAAATTGCAAA |
| LST1-fw | ACAAGAGAGGCACCAAGGAG | 62 |
| LST1-rev | GCTCGAGATGGACTGAGAGG |
| LTA-fw | CATGACACCACCTGAACGTC | 62 |
| LTA-rev | TGCTCAAGGAGAAACCATCC |
| MLSTD1-fw | AGTACTGGAATGCGGTCAGC | 62 |
| MLSTD1-rev | GCGCACGTCAAAGTTGAATAC |
| MMP7-fw | GACATCATGATTGGCTTTGC | 62 |
| MMP7-rev | GCCAAGTTCATGAGTTGCAG |
| PBX3-fw | GCAGCCTCTGGAGGTTCTTC | 60 |
| PBX3-rev | TTTGAGCTGCATCTGAATGG |
| PDE4D-fw | ACCGGCCCTTGACTGTTATC | 60 |
| PDE4D-rev | AGCAATTTAAAGCCCACAGC |
| PLEKHC1-fw | TCTGACCATGCTCTCTGGTG | 60 |
| PLEKHC1-rev | AGTGCCTCATCTTCAGACTGG |
| RB1-fw | TCCCATGGATTCTGAATGTG | 60 |
| RB1-rev | CCTTCTCGGTCCTTTGATTG |
| S100A4-fw | CAAGTACTCGGGCAAAGAGG | 62 |
| S100A4-rev | CTTCCTGGGCTGCTTATCTG |
| S100A10-fw | TGCCATCTCAAATGGAACAC | 62 |
| S100A10-rev | GCCCACTTTGCCATCTCTAC |
| 18S rRNA-fw | CGGCTACCACATCCAAGGAA | 60 |
| 18S rRNA-rev | GCTGGAATTACCGCGGCT |
| STK39-fw | CTCTGTGCACGACTCTCAGG | 62 |
| STK39-rev | GAGCAAACCCAATCAGCTTC |
| TMEM45A-fw | TGCGGTCAAGTCTCATTCTG | 62 |
| TMEM45A-rev | TCCAACTTCTGAGGAGCAGAG |
